# Solubility and Metastable Zone Width Measurement of Na_2_CO_3_ Hydrate Phases in the Na_2_CO_3_–NaOH–H_2_O System as a Basis for a Novel Carbon-Negative Soda Ash Production Strategy

**DOI:** 10.1021/acs.iecr.5c00320

**Published:** 2025-05-12

**Authors:** Somayyeh Ghaffari, Maria F Gutierrez, Peter Schulze, Andreas Seidel-Morgenstern, Heike Lorenz

**Affiliations:** 28307 Max Planck Institute for Dynamics of Complex Technical Systems, Magdeburg 39106, Germany

## Abstract

This study supports a carbon-negative soda ash production process by integrating CO_2_ direct air capture in NaOH solutions with crystallization. It presents solid–liquid equilibrium (SLE) and metastable zone width (MSZW) data for Na_2_CO_3_ hydrates in the Na_2_CO_3_–NaOH–H_2_O system, relevant to the so-called CODA process. Due to limited previous SLE data, new solubility models were developed. SLE data were determined for decahydrate (3.2–17.1 °C, 0–10 wt % NaOH) and monohydrate (35–85 °C, 0–15.7 wt % NaOH), and concentrations were measured by pH-titration with automated dosing. To prevent undesired primary nucleation in future industrial processes, MSZW was determined for both hydrates for the first time: by cooling crystallization for decahydrate and vacuum evaporative crystallization for monohydrate. Results show that decahydrate exhibits a wide, stochastic MSZW (Δ*T*_max_: 20.9–3 K), narrowed by lower temperatures but widened by NaOH. In contrast, monohydrate has a comparatively small MSZW (average supersaturation ratio: 1.08), independent of both evaporation rate and NaOH presence.

## Graphical abstract

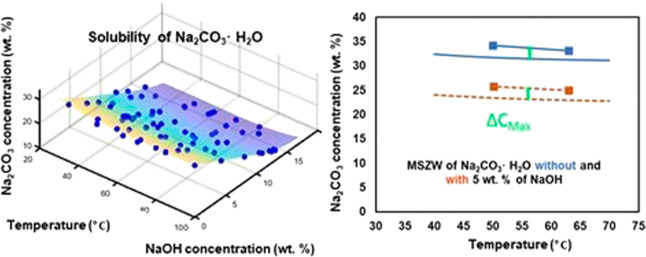

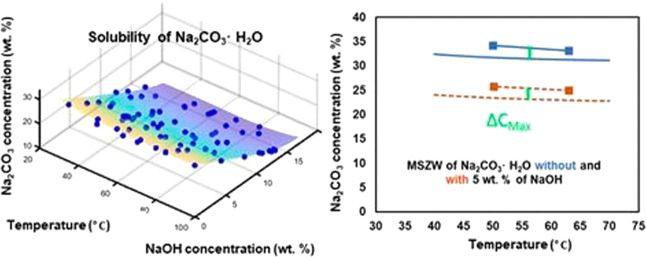

## 1 Introduction

Sodium carbonate production was historically realized from plants such as seaweed and barilla. The amount of sodium carbonate extractable from seaweed (kelp) ranges from only 2 to 3 wt %, whereas barilla can contain up to 30% sodium carbonate. Sodium carbonate can also be produced from minerals like trona (Na_2_CO_3_·NaHCO_3_·2H_2_O), which contains 70.39% Na_2_CO_3_. The United States, China, and Turkey possess the largest trona reserves globally. The industrial production of sodium carbonate was first established in 1790 through the Leblanc process. However, this process became obsolete due to the emission of HCl and CaS, as well as its low yield.^1^ Nowadays, the ammonia-soda (Solvay) process is the most common method for producing sodium carbonate, with more than 90% of sodium carbonate in Europe being produced via this way. However, due to the high environmental impact of this process, such as CO_2_ and NH_3_ emissions and the release of wastewater containing CaCl_2_, it should be replaced by new methods.

The principles and initial key parameters for replacing this conventional process with an environmentally friendly and sustainable alternative were presented with a focus on the DAC CO_2_ reactive absorption process in NaOH solutions within the CODA (Carbon-negative sODA ash) project.^2^ An experimental and modeling study of a newly designed droplet absorber for CO_2_ DAC was also conducted.^3^ Recently, in the related project, various process variants were investigated combining the reactive CO_2_ absorption in aqueous NaOH to produce a Na_2_CO_3_ solution with subsequent crystallization of either Na_2_CO_3_ decahydrate followed by the monohydrate, or directly of the monohydrate, thus exploiting either two or only one crystallization stage(s).^4^ The anhydrous Na_2_CO_3_ (soda ash) product is ultimately produced by dehydrating the monohydrate to generate the sodium carbonate with high bulk density (heavy soda ash, >1100 kg/m^3^) as desired by e.g., the glass industry as main soda ash user.

Phase behavior and solubility in the Na_**2**_ **CO**_**3**_-NaOH-H_**2**_O system. As partly already mentioned, sodium carbonate forms at least three stable hydrates, the mono-, hepta-, and decahydrate. Besides, the existence of other stoichiometric hydrates containing 1, 1 $\frac{1}{2}$ , 2, 2 $\frac{1}{2}$ , 3, 4, 5, 6, 7, 8, and 9 molecules of water per molecule of sodium carbonate has been questioned by various authors, another hydrate, Na_2_CO_3_·15H_2_O, has also been described, at very low temperatures.^5^ The existence of a hydrate may be detected by observation of a transition point from the solubility curve or by other methods such as the dilatometer method, but it still may remain a great difficulty to isolate and characterize the indicated hydrate. An author^5^ has discussed other methods for isolating all hydrates of a salt and investigated a method for hydrate isolation. This method was based on the fact that a hydrate is dehydrated by a lower hydrate until the hydrate next in the series to the lower hydrate is formed. As a case study of this method only the mono-, hepta- and decahydrate of sodium carbonate were isolated, and no intermediate hydrate was isolated. The monohydrate and the decahydrate are well-known commercial products. Sodium carbonate monohydrate is commercially important as the precursor of dense soda ash, which is widely used in the chemical and allied industries. The monohydrate is stable in the presence of saturated solution only above 308 K, and the decahydrate is stable at room temperature. In air, however, the decahydrate loses water to finally form the monohydrate, which is sufficiently stable to have been suggested as an acidimetric standard.^6^

Both temperature and NaOH concentration affect the transition point of the hydrates, which has been demonstrated within the temperature range of 5–120 °C and the NaOH concentration range of 0 to approximately 50 wt % for all mono-, hepta-, and decahydrates.^7^ As the concentration of NaOH increases, the transition temperature between phases occurs at lower temperatures. In this study, the effect of the NaOH concentration on the phase transition at a temperature of 10 °C, the average temperature in Germany,^8^ is examined.

Old literature sources,^7,9–16^ with all their data compiled in,^17^ report the SLE data of Na_2_CO_3_, NaOH, and H_2_O system across a wide range of NaOH concentrations and temperatures. However, there is insufficient data for the CODA process conditions, which are around 5 wt % NaOH, the optimal concentration for DAC CO_2_ capturing.^2^ In this work, SLE data were obtained for NaOH concentrations ranging from 0 to 12 wt % and temperatures from 3 to 17 °C for decahydrate, and for NaOH concentrations from 0 to 15 wt % and temperatures from 35 to 85 °C for monohydrate. The concentrations of Na_2_CO_3_ and NaOH for the SLE measurements were analyzed using an automated titration device, based on the pH change method. This device is sensitive to pH changes in the dosing of added acid, ensuring high accuracy. Solid phases were also analyzed with the presented titration method. Subsequently, both the literature data and the newly obtained experimental data were used to develop a polynomial fit for both decahydrate and monohydrate.

Within the metastable zone, a solution is supersaturated but has not yet undergone spontaneous nucleation within a certain finite time. This zone lies between the solubility line and a hypothetical line, where spontaneous nucleation occurs. This line is a kinetic value and depends on various experimental conditions. The most influential parameters include the rate of supersaturating the system, such as the cooling rate and evaporation rate of the solvent, but also the concentration of additives or impurities present, the scale and the thermal history of the solution—specifically, how long and to what degree the phase has been heated above saturation.^18^ Thus, the metastable zone width (MSZW) is a characteristic of the system and can vary between laboratory and plant scales, although there are cases where no variation was observed. It can also be independent of the technique used to generate supersaturation, whether through cooling or evaporative crystallization.^19^ In this work, the MSZW of the Na_2_CO_3_ decahydrate is measured using cooling crystallization in both small laboratory and 3L-scale, and that of the monohydrate using vacuum evaporative crystallization at constant temperature and constant evaporation rate in the 3L-scale. The scale of 3L was also applied for the subsequent crystal growth study, allowing for the collection of sufficient samples for analysis. In addition, it more closely resembles industrial conditions. To our knowledge, there has been no prior research examining the MSZW of Na_2_CO_3_·10H_2_O and Na_2_CO_3_·H_2_O in the Na_2_CO_3_–NaOH-H_2_O system.^18^ reports the MSZW of Na_2_CO_3_·10H_2_O only at the equilibrium temperature of 28 °C through cooling crystallization, and classifies it as a substance that does not crystallize spontaneously. Therefore, with respect to the crystallization of the Na_2_CO_3_ deca- and monohydrates in the novel CODA process, the MSZW of these hydrates was measured over a wider temperature range and in absence/presence of NaOH. In addition, since the decahydrate might crystallize already in a droplet absorber, the influence of gas bubbles was considered.

In the following, after describing the materials, setups and experimental and modeling methods used, first, the solubility data in the Na_2_CO_3_–NaOH-H_2_O system obtained both experimentally and through modeling, and second, the MSZW data determined for Na_2_CO_3_·10H_2_O and Na_2_CO_3_·H_2_O as mentioned above are shown and discussed.

## 2 Experimental Tools and Methods–Description and Evaluation

### 2.1 Materials

NaOH (pellets), Na_2_CO_3_, and Na_2_CO_3_·10 H_2_O, all with ≥99.0% purity were purchased from Merck, Germany. Na_2_CO_3_·1H_2_O, with a purity of ≥99.0% was purchased from Sigma-Aldrich, Germany. Deionized water from a Milli-Q system by Merck, Germany was used in all experiments.

### 2.2 Apparatuses

The Eco-Titrator Acid/Base from Metrohm/Switzerland is used for analyzing concentrations of aqueous solutions of Na_2_CO_3_ and NaOH. It allows for programming based on equivalence points detected through pH changes. To ensure accuracy, titrations were conducted in a 150 mL closed-cap cylinder, preventing NaOH from reacting with CO_2_ in the air. The automated dosing rate of this device can be as low as 0.02 mL/min, enabling high accuracy in equivalence point detection.

For measuring the MSZW of Na_2_CO_3_·10H_2_O in a lab scale, the multiple reactor setup Crystalline from Technobis/The Netherlands was used. It combines temperature and transmissivity measurements with real time particle imaging at the smallest scale, as low as 2.5 mL. Transmissivity measures how much light passes through a substance without being absorbed or scattered, and is expressed as a percentage, indicating the proportion of light that successfully passes through the fluid. Eight glass reactors with both magnetic and overhead stirrers are available. The setup allows for control and observation of crystallization processes.

The Turbidity Transmitter TRB 8300 from Mettler Toledo/Switzerland was utilized to detect the nucleation point within the cooling and evaporative crystallization experiments in a 3L-scale. Turbidity measures the cloudiness or haziness of a fluid caused by a high concentration of suspended particles. It is typically measured in Nephelometric Turbidity Units (NTU) using a nephelometer, which assesses the scattering of light as it passes through the fluid. Turbidity measurement is commonly used to monitor the increasing concentration of particles (crystals) in a process liquid.^20,21^ In this work a relative scale, indicated in percentage (%), was employed. The sensor was installed directly in the crystallizer. Before crystallization in a clear medium, the offset in the manual calibration menu is adjusted to 0%. As nucleation starts, the measurement value increases. To enhance sensitivity in detecting the onset of nucleation, the slope should be adjusted to a higher value.

The Vacuum pump PC 3001 VARIO, Vacuumbrand/Germany, was used to provide the vacuum conditions needed for the evaporation crystallization of monohydrate.

To detect the nucleation point within vacuum evaporative experiments, attenuated total reflection Fourier transform mid-infrared (ATR-FT-MIR) spectroscopy was applied to measure the spectra of mixtures of Na_2_CO_3_, NaOH, and H_2_O inline. For this purpose, the ReactIR 45m equipped with a fiber optic probe from Mettler Toledo/Switzerland was utilized. The silver-halide optical fiber and the diamond optical window allow for the collection of spectra in the wavenumber range of 1900 to 650 cm^–1^. In this range, for the used ternary mixture two bands occur at 1637 and 1368 cm^–1^. The peak at 1640 cm^–1^ is associated with the bending vibration of water, while the wavenumber of 1368 cm^–1^ with the carbonate ion (CO_3_^2–^). This specific wavenumber can be used to detect the change in amount of carbonate groups. As the nucleation starts, and Na_2_CO_3_ hydrate crystals start to grow, the intensity of this wavenumber decreases.

### 2.3 Experimental Methods

#### 2.3.1 Solubility Measurement with Excess (Isothermal) Method

This method^19^ is widely used and involves the following steps: (1) Sample preparation and sample pretreatment: the measurement was performed in a closed sample vessel to prevent the loss of water through evaporation and the absorption of CO_2_ from the air. The solvent, water (with or without NaOH), and an excess amount of the Na_2_CO_3_ were introduced into a 50 mL sample vial. For sample pretreatment, all solid material was dissolved by heating above the expected saturation temperature and subsequently recrystallized by preferably fast cooling in order to produce finely dispersed particles that are well distributed in the solution. It was also necessary to seed with the expected hydrate (mono- or decahydrate). (2) Equilibration and stirring at constant temperature: the sample was kept inside a temperature-controlled jacket for at least 2 days to reach equilibrium at a constant temperature. Generally, establishment of equilibrium can be verified by observing a constant concentration over time. In our specific system, this was confirmed using an FTIR probe in solution of a 15% suspension density at 3 °C (the minimum temperature used), demonstrating that 2 days is sufficient to reach equilibrium. (3) Separation of phases: the solid and liquid phases were separated via vacuum filtration immediately after removing the sample vessels from the temperature-controlled jacket. Filters were kept in the oven/refrigerator for at least half an hour to match the temperature of the solubility measurement vessel. The concentrations of ions in the liquid phase were obtained using the titration method and moreover, also the solid phase was identified via the titration method.

#### 2.3.2 Solid Phase and Liquid Phase Analysis Method–Titration

##### 2.3.2.1 Liquid Phase Analysis

The titration method using a pH meter was employed to measure the concentration of the analyte, i.e., an aqueous solution of Na_2_CO_3_, with or without NaOH. Hydrochloric acid (HCl) served as the titrant, and the reactions occurred as shown in reactions 1–3 below. Consequently, the entire titration process has three equivalence points (EP_1_, EP_2_, and EP_3_) or breaks on the pH curve corresponding to the three stages (see Figure 1). The pH range of each EP is mentioned in the following reactions. For the solutions without NaOH there are only two stages. The Eco-Titrator was programmed according to the obtained values of the EPs, which represent the volume of acid used to reach each EP, and the stoichiometry of the reactions. The formulas to calculate the concentrations of NaOH and Na_2_CO_3_ are given in the Supporting Information. The only input at the Eco-Titrator for each measurement is the weight of the mixture solution, which was typically diluted with Milli-Q water according the expected concentration. The volume of the entire analyte should be sufficient to immerse the electrode in the solution, yet small enough to complete the titration within the confines of the closed cap cylinder.

R1 $$\mathrm{NaOH}\left(\mathrm{aq}\right)+\mathrm{HCl}\left(\mathrm{aq}\right)\to \mathrm{NaCl}\left(\mathrm{aq}\right)+H_{2}O\left(l\right)\left(\mathrm{EP at a pH}∼11\right)$$

R2 $${\mathrm{Na}}_{2}{\mathrm{CO}}_{3}\left(\mathrm{aq}\right)+\mathrm{HCl}\left(\mathrm{aq}\right)\to {\mathrm{NaHCO}}_{3}\left(\mathrm{aq}\right)+\mathrm{NaCl}(\mathrm{aq})\left(\mathrm{EP at a pH}∼7\right)$$

R3 $${\mathrm{NaHCO}}_{3}\left(\mathrm{aq}\right)+\mathrm{HCl}\left(\mathrm{aq}\right)\to \mathrm{NaCl}\left(\mathrm{aq}\right)+{\mathrm{CO}}_{2}\left(g\right)+H_{2}O\left(l\right)\left(\mathrm{EP at a pH}∼3\right)$$

**Figure 1:**
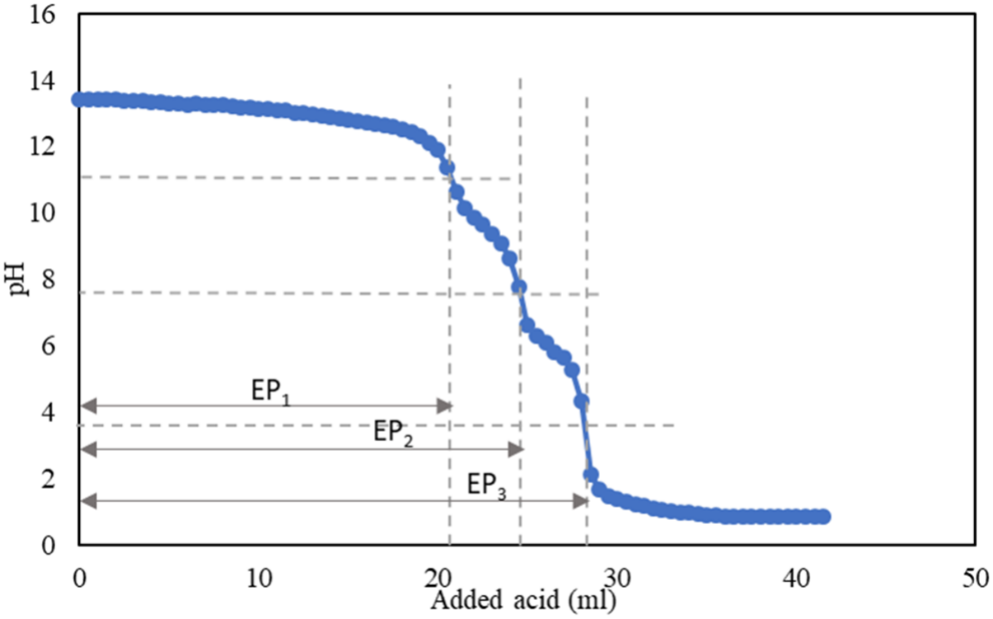
Typical pH change during titration of an aqueous mixture of NaOH and Na_2_CO_3_ with HCl.

When detecting equivalence points using indicators instead of a pH meter, reactions 1 and 2 can be identified by phenolphthalein, while reaction 3 can be indicated by methyl orange. In this scenario, for obtaining all EPs, it is assumed that EP_3_-EP_2_ is equal to EP_2_-EP_1_. However, using the Eco-Titrator allows for the independent determination of all EPs, revealing a slight difference between them. Though, in this paper, the average values of EP_2_-EP_1_ and EP_3_-EP_2_ were used for calculating the Na_2_CO_3_ concentration. It was found that using this average provides more robust and repeatable data, compared to using only EP_2_-EP_1_ value, for calculating the concentration of Na_2_CO_3_.

##### 2.3.2.2 Solid Phase Analysis

To analyze the solid phase for SLE measurements, assuming there is no intermediate hydrate, the titration method was used. In this method, the hydrate of Na_2_CO_3_ is dissolved in a specific amount of water, and the amount of Na_2_CO_3_ within the Na_2_CO_3_ hydrate is measured via titration. Since the amount of water added to dissolve the hydrate is known, the number of water molecules in the hydrate can be calculated. This method was first tested on reference hydrates where the substance bottles were newly opened, and the results showed that the titration method could identify, on average, 9.71, 0.925, and −0.02 molecules of water for the decahydrate, monohydrate, and anhydrous forms, respectively. Since the solid samples obtained from SLE measurements are wet and contain NaOH, the method was precisely examined by analyzing the reference samples after separation as follows: the solid samples were first immersed into saturated solutions of NaOH and Na_2_CO_3_ at ambient temperature, then filtered and washed with ethanol to remove the saturated solution, and finally dried to analyze the hydrate without excess solution using titration. In the preliminary tests with known hydrates, it was found that rinsing twice with ethanol while applying a vacuum filter and drying for 1 h by exposing to the air allows the method to detect the expected number of water molecules in the reference samples. Therefore, the solids from the SLE measurements were analyzed using the same method. It should be noted that the drying time for decahydrate should not be extended, as it can lose hydrate water quickly at room temperature.

#### 2.3.3 Metastable Zone Width (MSZW) Measurement

The MSZW in aqueous solutions can be experimentally determined by either the isothermal or polythermal method. The isothermal method essentially involves measuring the induction period *t*_ind_ for various supersaturated solutions. Isothermal methods described in the literature are all based on placing a defined supersaturated solution at a higher temperature in a cuvette or closed ampule and rapidly cooling it to the required saturation temperature *T*_eq_ and measuring the time elapsed from reaching *T*_eq_ to a change in the state of the solution. The solution can either be stirred or not during the measurement. In the polythermal method the solution is cooled in a controlled manner from the saturation temperature or higher down to the temperature at which visible crystals appear or where there is a discontinuity in the temperature dependence of the conductivity, refractive index, etc. The difference in these temperatures is the maximal supercooling, Δ*T*_max_.^18^ Then, finally these Δ*T*_max_ values are plotted as a function of the cooling rate and provide, extrapolated to a fictive cooling rate of 0 K/min, the Δ*T*_max,0_ value as a measure of the MSZW independent of a cooling rate applied. In this work, the MSZW for Na_2_CO_3_·10H_2_O was measured with the polythermal method at both 4 mL- and 3L-scales. Since the solubility of Na_2_CO_3_·1H_2_O has small dependency on temperature, and for crystallization from solution vacuum evaporation will be utilized, vacuum evaporation was also used to characterize the MSZW at the 3L-scale.

##### 2.3.3.1 Small-Scale Lab Apparatus

The Crystalline device was used to measure the MSZW of Na_2_CO_3_·10H_2_O. Approximately 4 mL of saturated solutions, whose concentrations were analyzed by titration, were placed inside the reactors. A top hook stirrer operating at 500 rpm was used for agitation. The sampling interval was 60 s. The analysis involved transparent particles that allowed light to pass through. After being agitated for approximately 1 h at 40 °C, the solutions were cooled at different rates to temperatures as low as −15 °C to detect the nucleation point. This point was identified by observing changes in temperature and transmissivity. Subsequently, the solutions were heated at different rates to determine the dissolution temperature, characterized by the first image with no crystals and 100% transmissivity. This procedure was repeated for at least three different cooling/heating rates to extrapolate the data to the zero cooling/heating rate, thereby determining the MSZW and the equilibrium temperature using the polythermal method. Experiments were conducted with both filtered and unfiltered solutions.

##### 2.3.3.2 3L- Scale

Figure 2 illustrates a schematic diagram of the setup used for conducting both cooling and vacuum evaporative crystallization experiments. This setup primarily consists of a 3L draft tube baffled crystallizer, a vacuum pump, a condenser, a balance for measuring condensed water, and analytic probes such as a temperature probe, an FTIR probe, a turbidity transmitter probe, and two thermostats for the condenser and for the temperature-controlled jacket of the crystallizer. The entire setup was sealed gastight to apply vacuum pressure. Real-time data from all analytic probes and the balance were recorded using a custom Python code.

**Figure 2:**
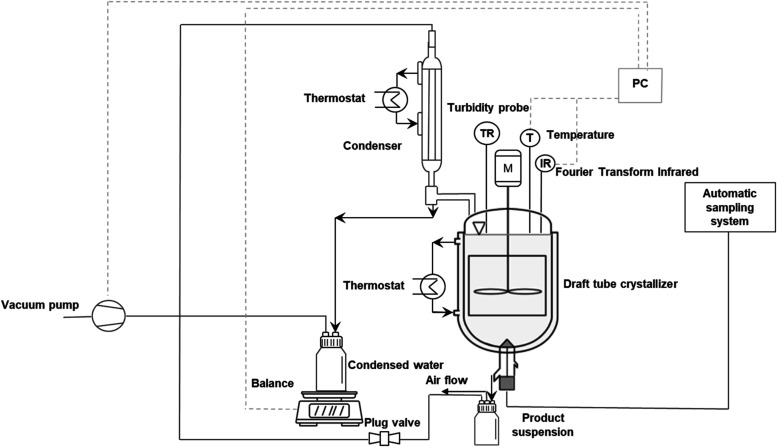
Process flowchart of the experimental 3L setup for the cooling and vacuum evaporative crystallization.

Batch cooling crystallization experiments for Na_2_CO_3_·10 H_2_O were conducted. Approximately 3 kg of feed solution was added to the crystallizer by applying vacuum pressure to draw the solution from the container into the reactor using a tube. The solution was stirred at 500 rpm for about 30 min at 30 °C; a temperature higher than the saturation temperature of all solutions used, and close to the temperature at which Na_2_CO_3_ has the highest solubility (32 °C). Subsequently, the liquor solution was cooled at rates ranging from 6 to 30 K/h using a thermostat that operated with thermal G fluid (Julabo) and could reach temperatures as low as −30 °C. The exact cooling rate was calculated from the internal temperature measurement inside the reactor, as reported in the Supporting Information, Table S13. Cooling continued until the nucleation point was reached detected by the turbidity probe. Additionally, at the nucleation point, due to the large metastable zone, the temperature increased sharply as crystallization is an exothermic process. This serves as another indication of the nucleation temperature. Figure 3 shows an example where both turbidity and temperature increase sharply at the same time as nucleation starts. The temperature just before this increase was chosen as nucleation temperature. Temperature and turbidity measurements were taken every 10 s. After nucleation, the suspension was heated to dissolve the crystals and determine the solubility temperature, at which the turbidity probe again provides 0% turbidity using different heating rates. Measuring the solubility with this method was done only for a few runs to validate the method and the analytics used; the results can be found in the SI.8. This procedure was repeated for different cooling/heating rates to extrapolate the nucleation and dissolution temperatures to the minimum cooling/heating rate, thereby determining the MSZW and solubility temperature, respectively.

**Figure 3:**
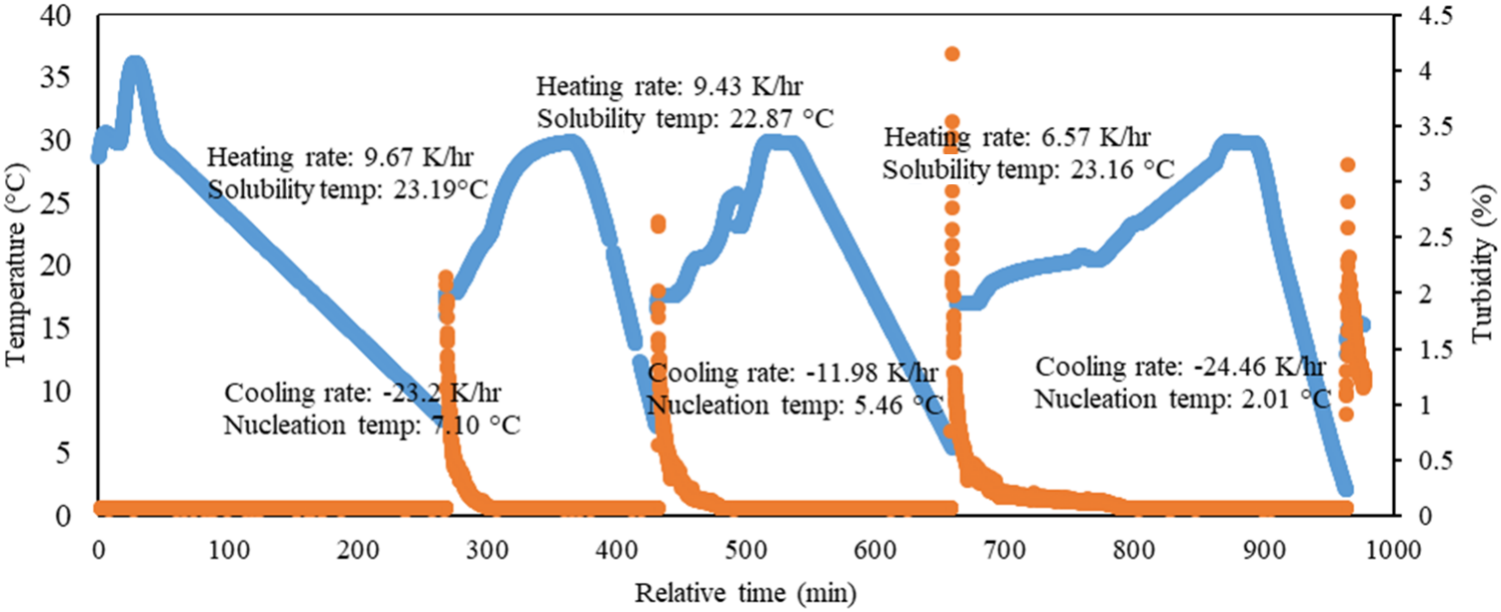
Recorded turbidity (orange points) and temperature (blue points) data for Run series 6 (SI.8).

The solubility of Na_2_CO_3_·1H_2_O is not significantly affected by temperature and this hydrate is stable at temperatures below the boiling point of its aqueous solution under atmospheric pressure. Therefore, vacuum evaporative crystallization at a constant temperature was employed to measure its MSZW at two temperatures, 50 and 63 °C. Approximately 3 kg of the feed solution was added to the crystallizer as previously described. The desired constant temperature for evaporative crystallization was maintained using a temperature-controlled jacket. To determine the corresponding saturation vapor pressure of the binary or ternary aqueous mixtures of NaOH and Na_2_CO_3_ at the desired temperature, the pressure was initially set to the saturation vapor pressure of pure water at the applied temperature. Subsequently, the pressure was gradually reduced until small evaporation bubbles formed inside the crystallizer. Once this pressure was identified as the saturation vapor pressure of the solution, the evaporation rate was adjusted by varying the external temperature in the reactor’s temperature-controlled jacket by a thermostat. At this point, the thermostat’s control factor should have been switched from external to internal to maintain a constant evaporation rate. The evaporation rate was calculated by continuously recording the data from the balance for condensate water. As evaporation commenced, the peak of the corresponding wavelength for the carbonate ion in the FT-IR spectra increased before reaching the nucleation point. Since the applied vacuum pressure is fixed, the temperature inside the reactor rises due to changes in saturation vapor pressure caused by concentration changes, leading to an increase in temperature inside the crystallizer. To maintain a constant temperature inside the reactor, the applied pressure was manually decreased periodically. Figure 4 illustrates how the temperature was manually controlled until the nucleation point by adjusting the applied vacuum pressure while maintaining a constant evaporation rate, as depicted in Figure 4 as well.

**Figure 4:**
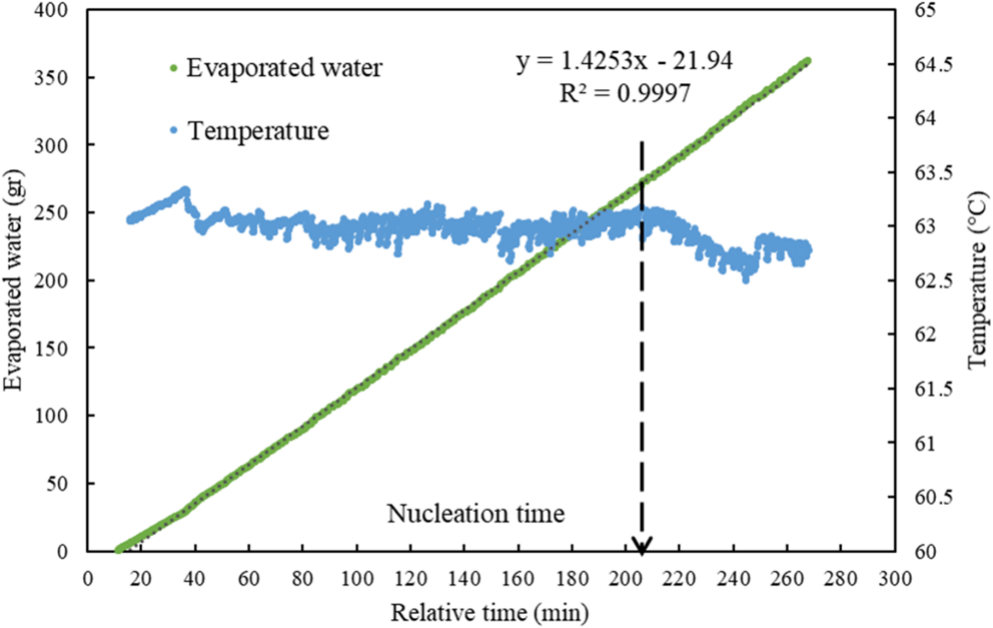
An example of the controlled constant temperature until the nucleation point, and the constant evaporation rate of water (Exp2.1, SI.11).

The nucleation point was detected using both turbidity and FT-IR probes, and an exemplary data plot is presented in Figure 5 for one of the experiments. FT-IR data were collected every 30 s, while turbidity data every 10 s. At the nucleation point, the concentration decreases as nucleation starts. The nucleation time measured with the FT-IR probe aligns with the time when turbidity increases. As shown in Figure 5, both probes exhibit some noise, possibly due to the presence of bubbles. After each experiment, the condensate water was poured back into the reactor to conduct the experiment with a different cooling rate by changing the external temperature in the reactor’s temperature-controlled jacket. An additional 7 g of water were also added to the reactor after each experiment to account for the average water loss mostly through the vacuum pump. It should be noted that the evaporation rate should not exceed certain limits to prevent excessive bubbles that could interfere with the analytical probes. A smooth evaporation process can also prevent the loss of dried carbonate on the walls due to large bubbles touching the walls. Therefore, the evaporation rates used were between 1 and 5 g/min, which corresponds to 0.007–0.036 wt % Na_2_CO_3_/min on this setup.

**Figure 5:**
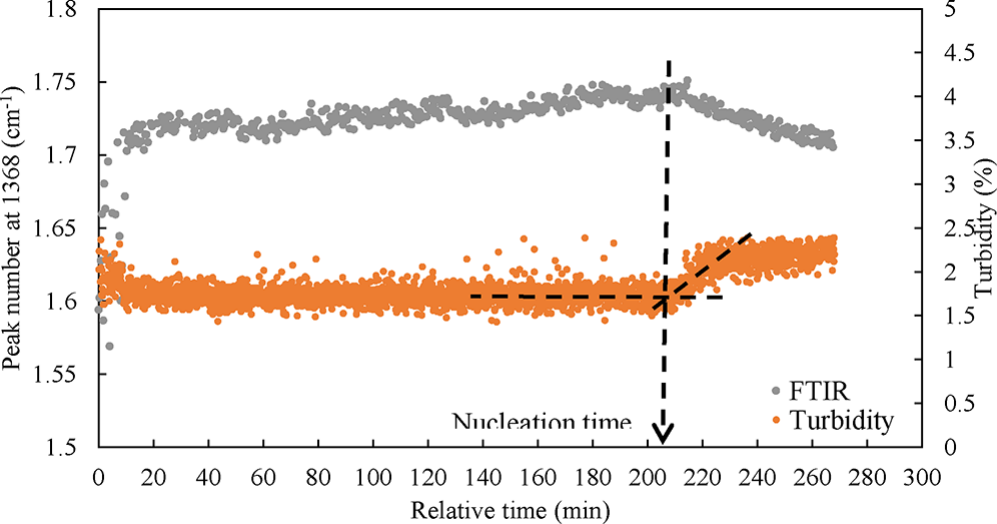
An example of detection of the nucleation point of Na_2_CO_3_.1H_2_O using FT-IR and turbidity probes in vacuum evaporation at constant temperature (Exp2.1, SI.11).

### 2.4 Modeling Methods

#### 2.4.1 Polynomial Modeling

The written MATLAB code, fits polynomial models of varying degrees (from 1 to 5) to the data set and evaluates their performance using the sum of squared errors (SSE) as the error metric. The polynomial with the lowest SSE if does not overfit, is selected as the best fit, indicating it has the smallest discrepancy between the predicted and actual values without overfitting. The obtained polynomial fits for each hydrate are compared with the following thermodynamic model as well.

#### 2.4.2 Thermodynamic Modeling

The solid–liquid equilibria of the Na_2_CO_3_–NaOH-H_2_O system were modeled based on the definition of the solubility product constant, where the solid compound is considered the reactant, and the ions formed in the liquid phase by dissolution of the ionic compound in water are considered the products. The dissolution reactions for sodium carbonate decahydrate and sodium carbonate monohydrate are presented in [Disp-formula eq4] and 5, respectively.

R4 $$Na_{2}CO_{3}\cdot 10H_{2}O↔2Na^{+}+{\mathrm{CO}}_{3}^{2-}+10H_{2}O$$

R5 $$Na_{2}CO_{3}\cdot H_{2}O↔2Na^{+}+{\mathrm{CO}}_{3}^{2-}+H_{2}O$$

The solubility product constant is an equilibrium constant (*K*) that relates the activity of the reactants and the products in the mixture at equilibrium, using the stoichiometric coefficients. For the compound in the solid phase, the activity was assumed to be one because both phases were supposed to be pure (no solid solutions).^22^ For compounds in the liquid phase, the activity was calculated as the multiplication of the mole fraction (*x*) and the activity coefficient (γ). The resulting equations to describe the solid–liquid equilibria are presented in eq 6 for the decahydrate and eq 7 for the monohydrate.

1 $$K_{Deca}=x_{Na^{+}}^{2}x_{CO_{3}^{2-}}x_{w}^{10}\gamma_{Na^{+}}^{2}\gamma_{CO_{3}^{2-}}\gamma_{w}^{10}$$

2 $$K_{\mathrm{Mono}}=x_{Na^{+}}^{2}x_{CO_{3}^{2-}}x_{w}\gamma_{Na^{+}}^{2}\gamma_{CO_{3}^{2-}}\gamma_{w}$$

The solubility product constants, *K*_Deca_ and *K*_Mono_, as a function of the temperature (*T* in K), were calculated with eq 8 and 9 for decahydrate and monohydrate, respectively. The coefficients of eqs 8 and 9 were taken from the Aspen Plus V12 database. These equations are equivalent to the thermodynamic expression that uses melting enthalpy, melting temperature and the change in heat capacity at melting to correlate phase equilibria.

3 $$\ln K_{\mathrm{Deca}}=15.6591-\frac{8896.83}{T}$$

4 $$\ln K_{\mathrm{Mono}}=-2270.32+\frac{66730.9}{T}+387.996\ln T-0.588063\times T$$

The solubility of sodium carbonate in the temperature range below 30 °C was calculated with eq 8 (decahydrate), while in the temperature range above 40 °C eq 9 was used (monohydrate). To take the presence of NaOH in the mixture into account, the mole fraction of the Na^+^ and OH^–^ ions (*x*_Na^+^_, *x*_OH^–^_) was considered in the activity coefficient calculation. The activity coefficient of the ionic compounds was determined with the electrolyte NRTL model (eNRTL) as explained in the literature.^22^ The eNRTL model was developed for the calculation of activity coefficients in aqueous and mixed solvent electrolyte systems.^23–25^ It was chosen over other electrolyte models because of its ability to describe the reactive gas–liquid equilibrium present in the CO_2_ absorption,^26^ which precedes the crystallization of Na_2_CO_3_ hydrates in the CODA process. The electrolyte system studied was composed of: H_2_O,Na^+^,OH^–^,CO_3_^2–^. The required ion–ion and molecule-ion interaction parameters were taken from the Aspen Plus V12 database and are reported in Tables S1–S4 in the Supporting Information. According to the model data bank, the parameters were obtained using the Aspen Physical Property Data Regression System (DRS) to regress vapor pressure and mole fraction data at 100 °C.

Depending on the hydrate, eq 6 or 7 is solved iteratively by first assuming a solubility concentration (*x*_Na^+^_ and *x*_CO_3_^2–^_) due to Na_2_CO_3_, and calculating the solubility product constant with eq 8 or 9 at a given temperature. The activity coefficients ((γ_Na^+^_,γ_*CO*_3_^2–^_),γ_w_) in eqs 6 and 7, are calculated via eNRTL model. This procedure is repeated until the calculated constant using eqs 6 and 7 agrees with the one obtained with eq 8 or 9. The equations were solved in Python and the calculation was validated with the results of a simulation of the solid–liquid equilibria in Aspen Plus V12. Figures S1 and S2 in Supporting Information present the validation of calculations.

When evaluating the eNRTL model, it was found that the parameters fitted by Gondal^26^ to describe the gas–liquid equilibrium are no longer able to describe the solid–liquid equilibrium. In contrast, the parameters on the database of Aspen Plus V12 were able to describe the solubility data in a much closer way (Figures S1 and S2 in the Supporting Information). Future work could be developed on finding proper pure and pair parameters able to describe both gas–liquid and solid–liquid equilibrium in the range of temperatures and concentrations in which the CODA process is carried out (5–100 °C and NaOH 0–15 wt %, 0–30% Na_2_CO_3_).

## 3 Results and Discussion

### 3.1 Solubility Measurements in the Na_2_CO_3_–NaOH–H_2_O System and Modeling

The effect of the NaOH concentration was studied at approximately 10 °C. At 9.9 °C, with NaOH concentrations below 15.44 wt %, only the Na_2_CO_3_ decahydrate was detected. At 19.03 wt % NaOH, heptahydrate, and at concentration of 30.47 wt % NaOH, monohydrate was observed. Detailed SLE data can be found in the Supporting Information, Table S5. The results align with the literature,^7^ which indicates that at 10 °C, the transition between the deca- and heptahydrate occurs at around 20 wt % NaOH (read from a figure), between the hepta- and monohydrate at approximately 20.27 wt % NaOH, and between the mono- and anhydrate at around 47.5 wt % NaOH.

Considering the effect of both NaOH concentration and temperature on transition between the Na_2_CO_3_ phases, the SLE data for the deca- and monohydrate were measured under conditions close to CODA conditions, together with solid phase analyses. For decahydrate, the SLE data were obtained in the temperature range of 3.2–17.1 °C and NaOH concentrations ranging from 0 to 10 wt %. The results are reported in Table 1. Literature data were selected for a temperature range of 5–30 °C and NaOH concentrations of 0–12.7 wt %, as shown in the Supporting Information, Table S6. For the monohydrate, the obtained SLE data cover the temperature range of 35–85 °C and NaOH concentrations of 0–15.7 wt %, and are compiled in Table 2. Literature data were selected for the temperature range of 30–100 °C and NaOH concentrations of 0–16.86 wt %, as presented in the Supporting Information, Table S7. The selected literature data represent only one solid phase. The experimental data, along with the literature data, were used to correlate a polynomial fit with two variables (temperature and NaOH concentration) for both the deca- and monohydrate phases. The polynomial fit equations and the obtained coefficients with a 95% confidence interval, Sum of Squared Errors (SSE) and correlation coefficients for each degree are reported for each hydrate in the Supporting Information, Figures S3–S8. The 3D graphs of the obtained fits are shown in Figures 6 and 7. Additionally, a polynomial fit is reported in the Supporting Information, Table S10, for the anhydrous phase using only literature data.^17^ Given the age of the literature data, it is improbable that an automated titration method, which enhances accuracy by adjusting the flow rate based on pH changes, was employed. Instead, indicators might have been used for the titration process. For the solid phase, however, the X-ray method was utilized, offering greater reliability than titration for detecting the solid phase, as titration does not accurately identify an intermediate hydrate. Nonetheless, by acknowledging the impact of NaOH concentration on the solid phase, the likelihood of an intermediate hydrate forming in our samples is minimal due to their low NaOH concentrations.

**Figure 6:**
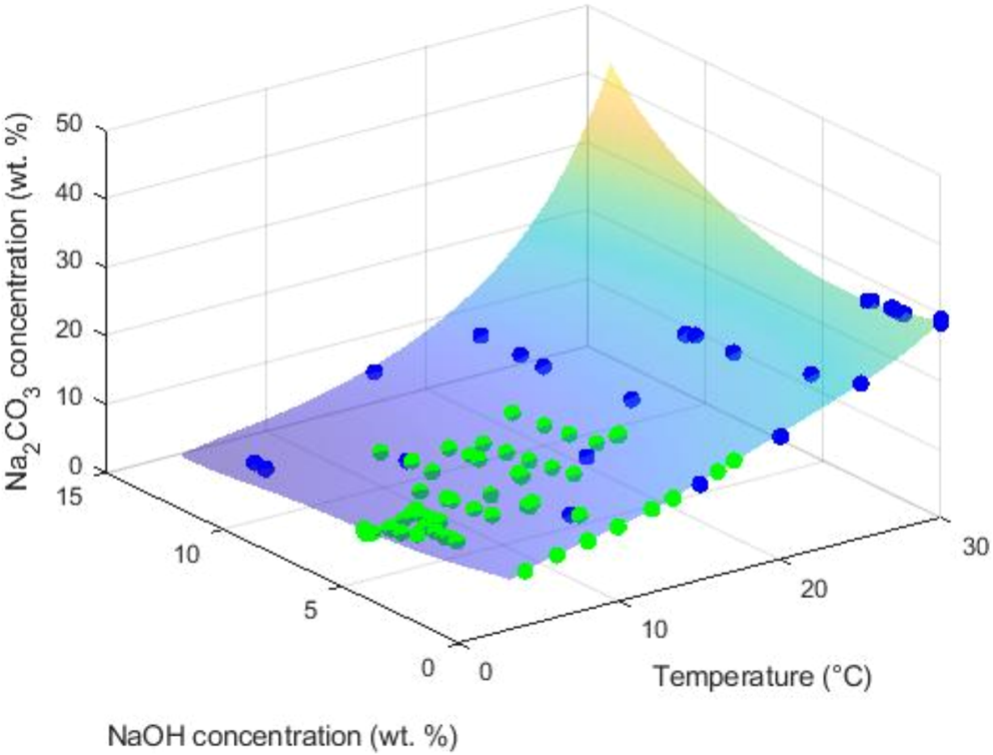
3D representation of the polynomial fit derived from own experimental (Table 1) (green color) and literature (Table S6) (blue color) SLE data for Na_2_CO_3_·10H_2_O in the Na_2_CO_3_–NaOH-H_2_O system. The polynomial fit is applied only within the region validated by data points.

**Figure 7:**
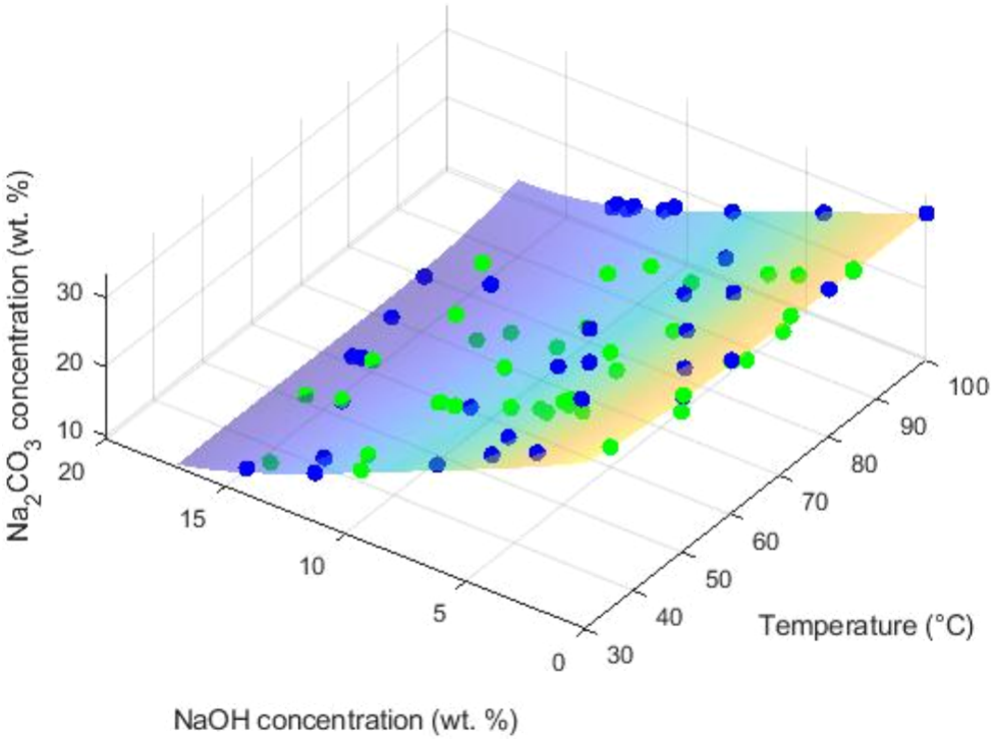
3D representation of the polynomial fit derived from own experimental (Table 2) (green color) and literature (Table S7) (blue color) SLE data for Na_2_CO_3_·1H_2_O in the Na_2_CO_3_–NaOH-H_2_O system. The polynomial fit is applied only within the region validated by data points.

**Table 1:** Measured SLE Data of Na_2_CO_3_·10H_2_O in the Temperature Range of 3.2–17.1°C, and a NaOH Concentration Range of 0–10 wt %^a^

sample number	*T* (°C)	*C*_NaOH_ (wt %)	*C*_Na2CO3_ (wt %)	sample number	*T* (°C)	*C*_NaOH_ (wt %)	*C*_Na2CO3_ (wt %)
1	3.2	5.87	4.45	33	9.9	3.65	8.47
2	3.2	6.16	3.9	34	9.9	3.76	7.74
3	3.2	6.23	4.4	35	9.9	3.86	7.65
4	4.1	0	8.09	36	9.9	5.41	6.64
5	4.1	4.57	5.85	37	9.9	7.88	5.91
6	4.1	5.22	4.98	38	9.9	8.75	6.03
7	4.1	5.62	4.8	39	9.9	10.09	5.09
8	4.1	5.65	4.86	40	12	0	12.32
9	4.1	5.76	4.81	41	12	5.49	8.01
10	5.2	3.99	5.65	42	12	5.54	7.83
11	5.2	4.21	5.46	43	12	5.66	8.08
12	5.2	4.54	5.65	44	12	7.33	7.37
13	5.2	5.04	5.37	45	12	7.71	7.37
14	5.2	5.93	4.98	46	12	8.6	6.87
15	5.9	5.84	6.28	47	13.3	0	13.1
16	5.9	5.91	6.02	48	13.3	4.19	9.64
17	5.9	6.04	5.24	49	13.3	5.11	9.02
18	6.1	0	9.18	50	13.3	6.09	8.48
19	6.1	4.97	5.75	51	13.3	7.05	8.08
20	6.1	5	5.74	52	13.3	8.03	7.66
21	6.1	5.15	5.73	53	16.1	0	15.26
22	6.1	5.38	5.51	54	16.1	5.14	10.86
23	6.1	5.62	5.45	55	16.1	6.3	10.25
24	8	0	10.1	56	16.1	7.35	9.74
25	8	0	10.15	57	16.1	8.71	9.34
26	8	4.05	7.13	58	17.1	0	16.27
27	8	4.81	6.77	59	17.1	0	16.26
28	8	5.74	6.53	60	17.1	0	16.29
29	8	5.96	6.38	61	17.1	4.86	11.9
30	8	7.1	5.52	62	17.1	4.89	11.97
31	9.9	0	11	63	17.1	4.9	12.04
32	9.9	1.63	9.99	64	17.1	4.96	11.67

a Analysis based on an automated titration method with a relative error of less than 1%.

**Table 2:** Measured SLE Data of Na_2_CO_3_·1H_2_O in the Temperature Range of 35–85°C, and a NaOH Concentration Range of 0–15.7 wt %^a^

sample number	*T* (°C)	*C*_NaOH_ (wt %)	*C*_Na2CO3_ (wt %)	sample number	*T* (°C)	*C*_NaOH_ (wt %)	*C*_Na2CO3_ (wt %)
**1**	35.1	0	33.11	21	59.9	14.93	11.31
**2**	35.1	10.09	18.07	22	63.1	0	30.19
**3**	35.1	14.15	11.36	23	63.1	5.69	23.47
**4**	49	6.96	21.52	24	70.5	0	30.18
**5**	49	9.29	18.55	25	70.5	4.57	23.97
6	49.7	0	30.14	26	70.5	9.4	14.91
7	49.7	4.12	24.45	27	70.5	12.75	11.34
**8**	49.9	4.75	24.39	28	72.1	0	31.56
**9**	49.9	5.65	22.11	29	72.1	8.47	18.23
**10**	49.9	5.91	22.28	30	72.1	11.65	13.02
**11**	49.9	4.63	25.38	31	72.1	13.94	12.54
**12**	49.9	10.16	17.32	32	84.5	2.18	27.58
**13**	49.9	14.17	12.23	33	84.5	6.66	20.28
**14**	50.1	0	32.32	34	84.5	10.14	16.77
**15**	50.1	5	24.44	35	84.5	15.35	11.13
**16**	50.1	10.07	17.38	36	85	0	31.12
**17**	50.1	15.72	10.65	37	85	0	30.89
**18**	59.9	0	31.78	38	85	3.54	25.52
**19**	59.9	4.77	23.79	39	85	8.44	19.92
**20**	59.9	9.45	17.84				

a Analysis based on an automated titration method with a relative error of less than 1%.

For the decahydrate, a fifth-degree polynomial fit was used because it had the minimum SSE without causing overfitting, as shown in the solubility curves in the Supporting Information Figure S5. For the monohydrate, a fourth-degree polynomial was used because a fifth-degree polynomial resulted in overfitting, as indicated in S9. The correlation coefficients of the polynomial fits were 0.9995 for the decahydrate and 0.9955 for the monohydrate, respectively. To evaluate the quality of the regression models, the correlation matrix and Variance Inflation Factor (VIF) values for temperature and NaOH concentration were analyzed for both the decahydrate and monohydrate cases. In the decahydrate system, the correlation between temperature and NaOH concentration was found to be −0.2439, indicating a weak negative relationship. The VIF values for both variables were 1.0633, confirming the absence of multicollinearity. For the monohydrate system, the correlation was 0.0301, suggesting an almost negligible relationship, with VIF values of 1.0009. These results indicate that multicollinearity is not a concern in either case, ensuring the stability and reliability of the regression models. As illustrated in Figure 6, the solubility of decahydrate is assessed over an extensive range; the solubility exhibits a significant dependency on temperature showing an increase with rising temperature. The presence of NaOH is observed to decrease its solubility. Figure 7 illustrates that, as already known, the solubility of the monohydrate exhibits a slight retrograde behavior with temperature, indicating that an increase in temperature leads to a decrease in solubility. Additionally, similar to the decahydrate, the presence of NaOH further reduces the solubility of the monohydrate. Figure 8 illustrates the solubility curves obtained with 5 wt % NaOH in the temperature range for both hydrates. For the decahydrate (Figure 8a), there is a deviation between the experimental and thermodynamic modeling results; for example, at temperatures below 12 °C, the deviation ranges from 1 to 1.3 wt %, which corresponds to approximately 7.2 tons of decahydrate in a 200 m^3^ crystallizer. The deviation between the polynomial fit obtained with only literature data and the polynomial fit from this work is in the range of 0.2–0.55 wt % at temperatures below 12 °C, corresponding to up to 3 tons of decahydrate. In the case of the monohydrate, as shown in Figure 8b, the thermodynamic model underestimates the saturation concentration. The solubility obtained with the polynomial fits increases from about 80 to 100 °C. To ensure accuracy, more experimental points at different NaOH concentrations should have been measured, but since this temperature range is outside the scope of the CODA project this has been neglected. The MSZW studied in this work is limited to the range of 50 to 63 °C. Only polynomial models were used in this work to address the MSZW. Although the use of polynomial solubility equations is restricted to the temperature and concentration ranges used for their fitting, they serve as a basis for mass balances of different CODA process variants within the project without the need for a thermodynamic model or a process simulator.

**Figure 8:**
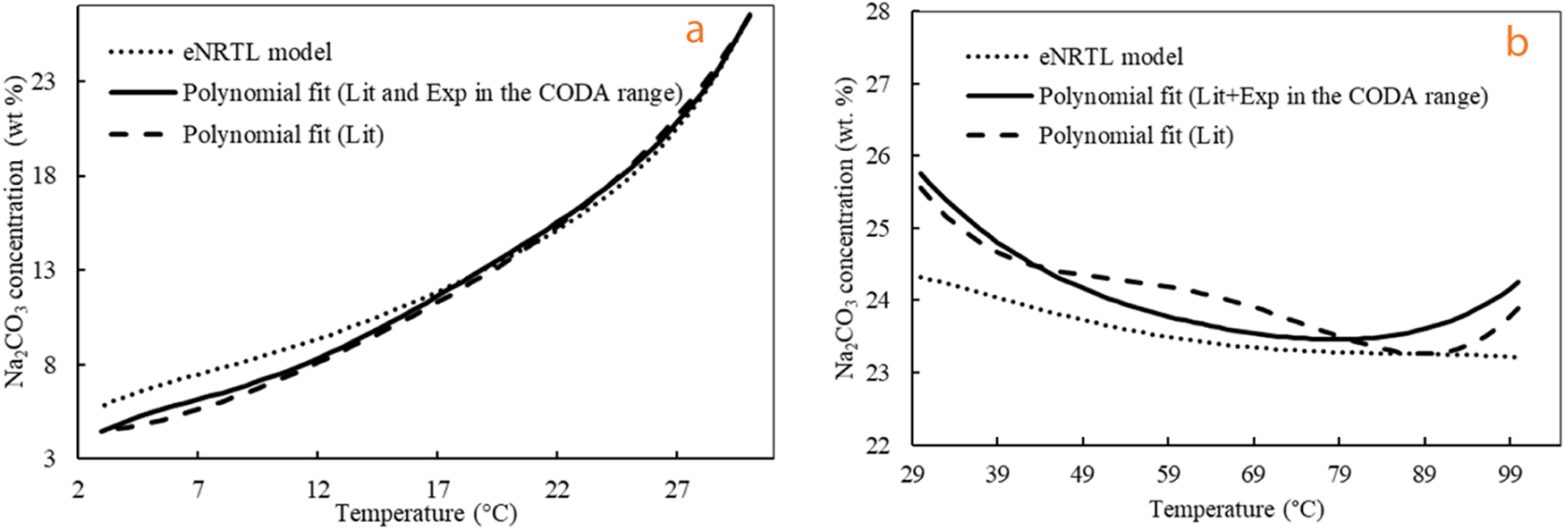
Solubility curve of Na_2_CO_3_ decahydrate (a), and Na_2_CO_3_ monohydrate (b), at 5 wt % NaOH concentration, obtained with the eNRTL model, the polynomial fit derived using the literature data, and polynomial fit of this work derived using both literature and experimental data in the CODA condition range.

### 3.2 Metastable Zone Width (MSZW) Measurements

#### 3.2.1 Small-Scale for Na_2_CO_3_·10H_2_O

Four samples with varying Na_2_CO_3_ concentrations without NaOH, and three samples with different Na_2_CO_3_ concentrations containing approximately 5 wt % NaOH, each with a volume of 4 mL, were filled into Crystalline reactors. The solutions were new and pure, and the saturation concentration of these samples corresponded to the equilibrium temperatures in the range from around 28 °C down to 9 °C. The cooling and heating rates ranged from 6 to 24 K/h. All solutions were double-filtered to remove particles larger than 0.2 μm. Detailed information on the experiments and the results can be found in the Supporting Information, Table S11. The results, presented in Figure 9, indicate that nucleation was more stochastic, as they do not follow the expected trend. It was expected that a higher cooling rate would result in a lower nucleation temperature. Consequently, the MSZW could not be precisely determined by extrapolating the nucleation points to the lowest cooling rate. However, the results reveal that NaOH widens the MSZW, and at lower temperatures, the MSZW is smaller than at higher temperatures. Δ*T*_max_ is found within the range of ∼32 K at higher temperatures to ∼12 K at lower temperatures without NaOH and within the range of ∼33 K at higher temperatures to ∼19 K to lower temperatures with around 5 wt % NaOH. Solubility data obtained using the polythermal method are also presented in Figure 9. These points are obtained by extrapolating the dissolution temperatures to the lowest heating rate, and as it is shown, there is only a slight difference from solubility data obtained with the isothermal method. No other research has studied the MSZW of Na_2_CO_3_·10H_2_O in the presence of NaOH. However, there is one study^18^ that reports the MSZW of Na_2_CO_3_·10H_2_O without NaOH at saturation temperature of 28 °C. Cooling rates of 2, 5, and 20 K/h were applied to 50 mL filtered solutions using a membrane filter with pore size of 0.28 μm. The obtained Δ*T*_max_ values were 17.77, 18.99, and 21 K, respectively. The study also examined the MSZW under the same conditions in the presence of crystals, where the MSZW decreased to 5.56, 6.30, and 7.60 K, respectively. These results are the largest MSZW among 33 other inorganic salts. For example, at the same cooling rates, the MSZW for Na_2_SO_4_·10H_2_O is 0.29, 0.39, and 0.64 K. The author claims that the obtained data are repeatable with their setup. However, when the same conditions were tested on Crystalline, the results were not replicable, with an average difference of 4.1 K. Other research works^27–29^ also showed that nucleation is a stochastic event, so that solutions of identical conditions nucleate at different, randomly distributed times, which is common for the homogeneous nucleation mechanism. For example, ice nucleation in the presence of 3–10 wt % NaCl was studied in Crystalline,^27^ and it was found that the nucleation occurs at different temperatures for the same cooling rate, with the first and last nucleation temperatures differing by 5–7 K for each of the compositions.

**Figure 9:**
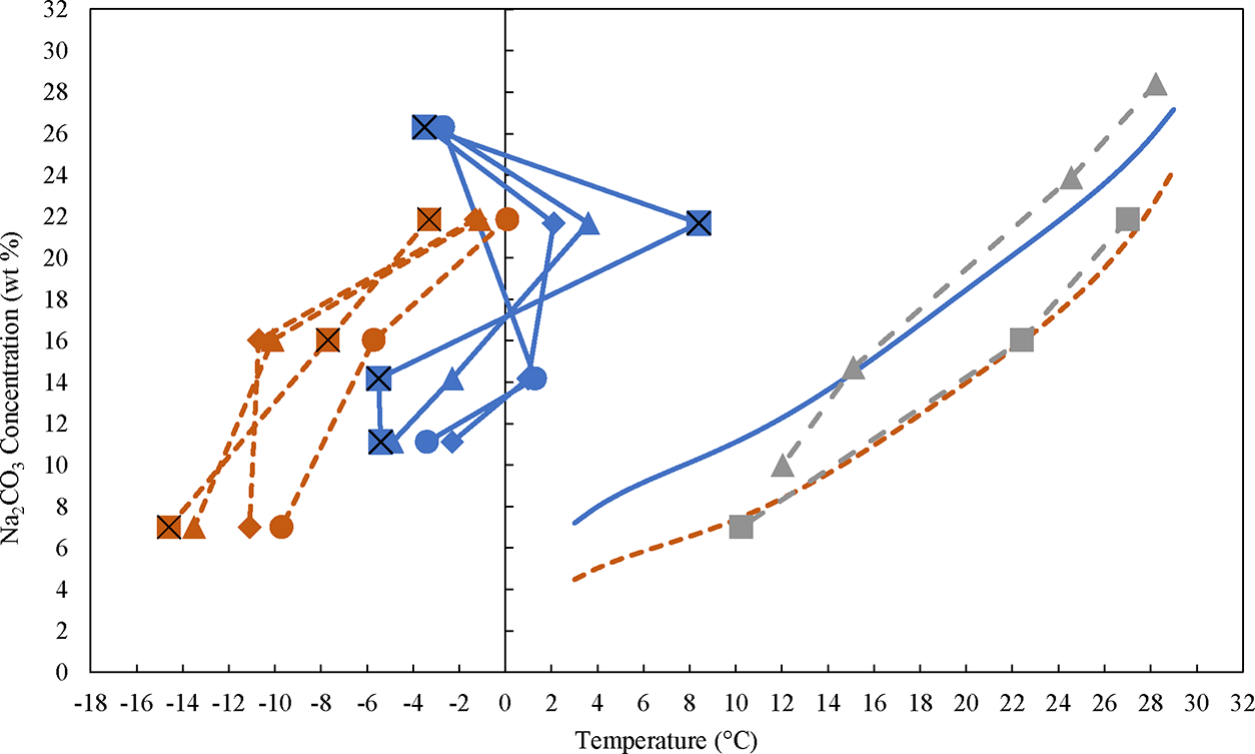
Solubility data obtained with the isothermal method from the polynomial fit without NaOH (blue solid line) and with 5 wt % NaOH (red dashed line). Solubility data obtained with the polythermal method in Crystalline without NaOH (gray dashed line with gray triangles) and with 5 wt % NaOH (gray dashed line with gray squares). The obtained nucleation points of Na_2_CO_3_·10H_2_O in Crystalline at different cooling rates in the range of 6–24 K/h, without and with 4.9 wt % of NaOH (particles bigger than 0.2 μm were removed). Nucleation points without NaOH for 6 K/h (blue solid line with blue circles), 12 K/h (blue solid line with blue diamond symbols), 8 K/h (blue solid line with blue triangles), and 24 K/h (blue solid line with blue cross symbols) Nucleation points with 5 wt % NaOH for 6 K/h (red dashed line with red circles), 12 K/h (red dashed line with red diamond symbols), 18 K/h (red dashed line with red triangle), and 24 K/h (red dashed line with red cross symbols).

Figure 10 shows the results of the same method and the same solution concentrations applied, but in this case, the solutions were not filtered to remove particles, allowing for comparison with larger-scale results, which is important for industrial applications. The cooling rates used ranged from 12 to 30 K/h. Detailed information about the experiments including results can be found in the Supporting Information, Table S12. The results illustrated in Figure 10 indicate that dust particles narrow the MSZW especially in the presence of NaOH. Δ*T*_max_ is within the range of ∼26 K at higher temperatures to ∼10 K at lower temperatures without NaOH and within the range of ∼32 K at higher temperatures to ∼14 K at lower temperatures with around 5 wt % NaOH. This clearly demonstrates, NaOH present widens the MSZW, and the lower the concentration, the smaller the MSZW.

**Figure 10:**
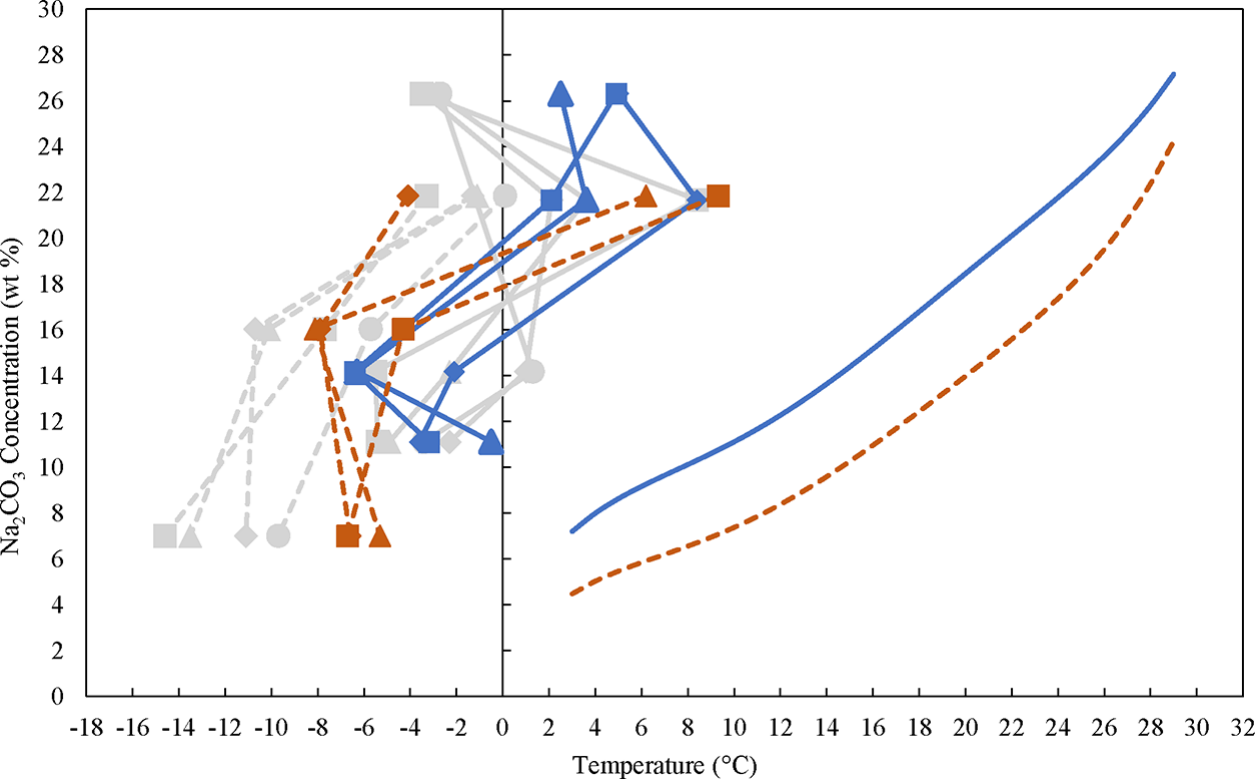
Solubility data obtained with the isothermal method from the polynomial fit without NaOH (blue solid line) and with 5 wt % NaOH (red dashed line). The obtained nucleation points of Na_2_CO_3_·10H_2_O from unfiltered solutions at different cooling rates in Crystalline. Nucleation points without NaOH for 12K/h (blue solid line with blue diamond symbols), 18 K/h (blue solid line with blue triangles), and 30 K/h (blue solid line with blue squares). Nucleation points with 4.9 wt % NaOH for 12K/h (red dashed line with red diamond symbols), 18 K/h (red dashed with red triangles), and 30 K/h (red dashed line with red squares). The gray background graphs are from Figure 9 for comparison.

#### 3.2.2 3L-Scale for Na_2_CO_3_·10H_2_O

Four solutions with varying Na_2_CO_3_ concentrations without NaOH, and three solutions with different Na_2_CO_3_ concentrations containing approximately 5 wt % NaOH, each with a volume of 3L, were used to measure the MSZW. The solutions were new and pure, with concentrations analyzed by the titration method. Using the polynomial fit obtained for the decahydrate (SI.3), the corresponding equilibrium temperatures were calculated. They were in the range of around 29.6 to 9.4 °C as reported in the Supporting Information, Table S13. The results obtained (detailed in SI.8) are presented in Figure 11. As can be seen, the MSZW gets much smaller in the bigger scale, and the lower the temperature, the smaller the MSZW. Δ*T*_max_ is found within the range of 17 K at higher temperatures to 3 K at lower temperatures without NaOH and within the range of 21 K at higher temperatures to 3 K at lower temperatures with around 5 wt % NaOH. This corresponds to a supersaturation ratio at nucleation point in the range of 2.29 at higher temperatures to 1.18 at lower temperatures without NaOH, and 4.36 at higher temperatures to 1.19 at lower temperatures in presence of 5 wt % NaOH. These results provide insights into preventing spontaneous nucleation in an industrial environment, which can pose challenges such as blockages in the units applied. For example, in one of the variants of the CODA process, the supersaturation for crystallization of Na_2_CO_3_·10H_2_O can be already achieved in the CO_2_ DAC using a droplet absorber.^3^ Working in a safe zone inside the MSZW is therefore very important in industrial applications.

**Figure 11:**
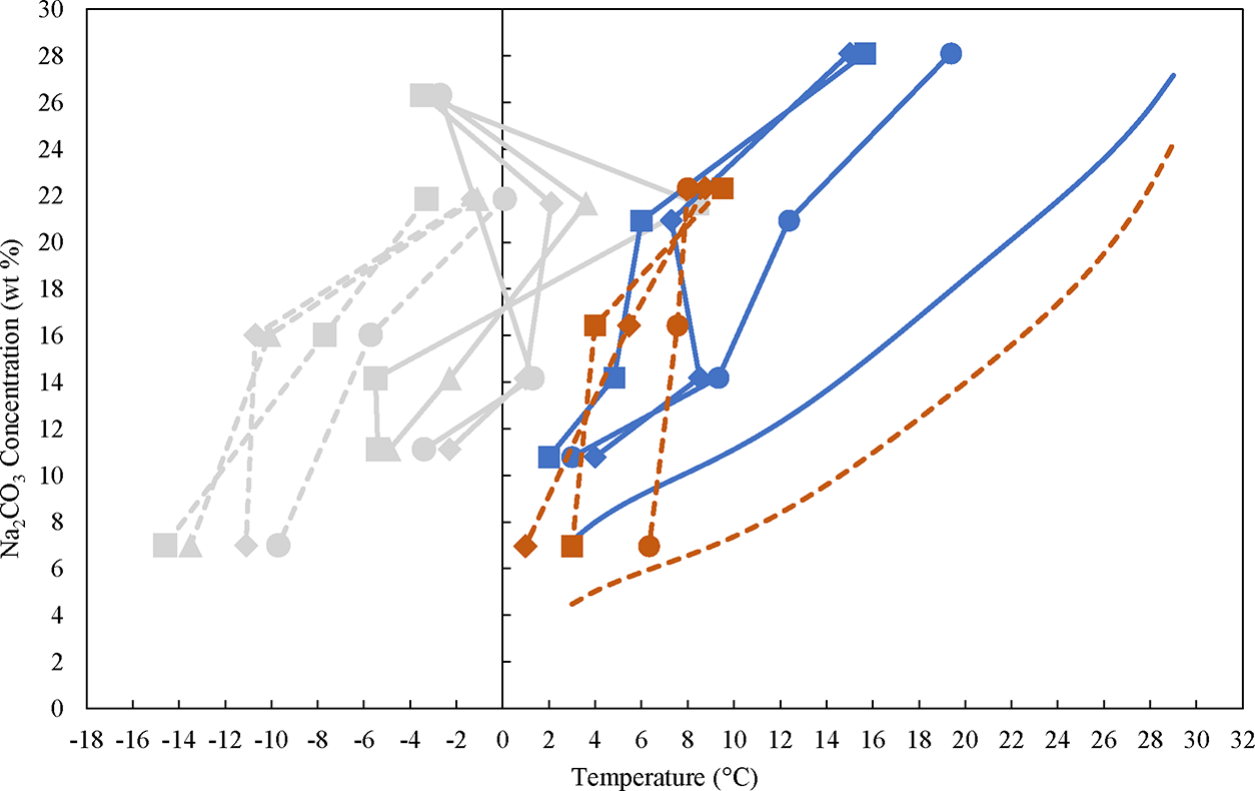
Solubility data obtained with the isothermal method from the polynomial fit without NaOH (blue solid line) and with 5 wt % NaOH (red dashed line). The obtained nucleation points of Na_2_CO_3_·10H_2_O in a 3L-scale. Nucleation points at different cooling rates without NaOH are shown for 6 K/h (blue solid line with blue circles), 12 K/h (blue solid line with blue diamond symbols), and 30 K/h (blue solid line with blue squares). Nucleation points with 5 wt % NaOH are shown for 6 K/h (red dashed line with red circles), for 12 K/h (red dashed line with red diamond symbols), and 30 K/h (red dashed line with red squares). The gray background graphs are from Figure 9 for comparison.

#### 3.2.3 3L-Scale for Na_2_CO_3_·10 H_2_O with Bubbles

In the CODA process, the CO_2_ DAC absorption process is combined with the crystallization process. As mentioned above, in one of the CODA process variants the absorber to be used in the absorption stage is a droplet absorber.^3^ For this reason, the MSZW was measured in the presence of bubbles within the same reactor at temperatures ranging from approximately 23 to 9 °C, using only 5 wt % NaOH to simulate the conditions of the droplet absorber. Synthetic air (air without CO_2_) was used to generate bubbles through a frit. The gas was introduced into the reactor at a rate of approximately 0.5 L/min using a tube equipped with a frit. The process flowchart of the experimental setup can be found in the Supporting Information, Figure S9. As depicted, the gas flows through a bottle of water to become saturated with water before entering the reactor. This step is performed to prevent frit blockage due to evaporation crystallization and to maintain consistent conditions throughout all experiments. Details of the experiments with the related results data are provided in the Supporting Information, Table S14. As derivable from the results depicted in Figure 12, the MSZW remains almost within the same range, 17.5–7 K. If this consistency is maintained in industrial applications, it is advantageous not to have a shortened MSZW, as this helps prevent unwanted primary nucleation in the event of a small temperature drop due to weather conditions.

**Figure 12:**
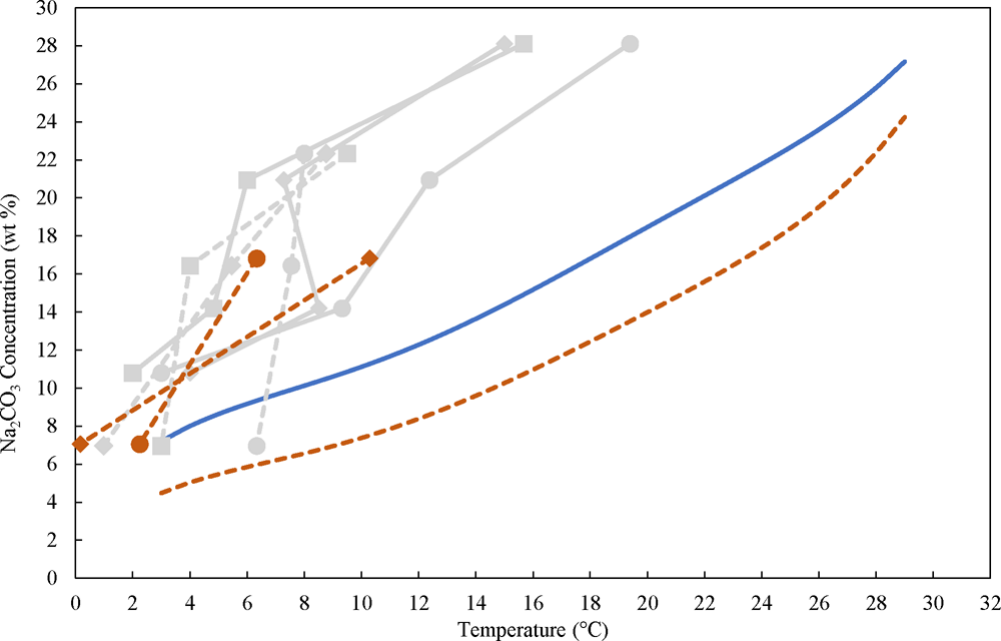
Solubility data obtained with the isothermal method from the polynomial fit without NaOH (blue solid line) and with 5 wt % NaOH (red dashed line). The obtained nucleation points of Na_2_CO_3_·10H_2_O in a 3L-scale with bubbling. Nucleation points at different cooling rates with 5 wt % NaOH for 6 K/h (red dashed line with red circles), and 12 K/h (red dashed line with red diamond symbols). Gray background graphs are from Figure 10 for comparison.

#### 3.2.4 3L-Scale for Na_2_CO_3_·1H_2_O

Since the monohydrate is stable in the temperature range around 32–105 °C (depending on the NaOH concentration), two new and pure solutions, each containing approximately 3 kg of Na_2_CO_3_, with and without NaOH, were evaporated at temperatures of 50 and 63 °C under vacuum pressures. These temperatures were chosen because lower temperatures require less energy, which is advantageous in industrial applications, since in the CODA process, the monohydrate is produced through vacuum evaporation and subsequently dried to produce soda ash. However, the temperature range was not set too low, i.e., too close to the phase transition temperature from the deca- to the monohydrate, to avoid the potential formation of an intermediate hydrate phase. Three different evaporation rates, ranging from 1.4 to 5.6 g/min, were applied. Detailed information on each experiment and the connected results is provided in the Supporting Information, Table S15. The concentrations of NaOH and Na_2_CO_3_ at the nucleation point were calculated based on the initial concentrations determined by titration and the amount of condensed water at the nucleation point. Corresponding saturation concentrations of the nucleation points are derived from the polynomial fit for monohydrate as presented in SI.4. The results data presented in Figure 13 show that the MSZW is almost independent of the evaporation rate, which is why an average MSZW value was calculated across different evaporation rates and is summarized in Table 3. As seen, due to evaporation, at the nucleation point the concentration of NaOH increases from initially 5 wt % to approximately 5.4 and 5.6 wt % for runs 1.1–1.3 and 1.4–1.6. Therefore, the reported MSZW is calculated based on the increased amount of NaOH. Figure 14 shows the MSZW of Na_2_CO_3_·1H_2_O in terms of concentration as a function of temperature for the temperatures applied. As from Figure 13, it becomes obvious that the MSZW of the monohydrate is independent of the presence of NaOH and only weakly affected by temperature in the range applied. Specifically, at a temperature of 50 °C, the MSZW is slightly larger than at 63 °C. In all of the experiments the supersaturation ratio ranged between only 1.06–1.12, and Δ*c*_max_ in the range of 1.72–2.76 wt %, as presented in the Supporting Information, Table S15. This provided us with greater confidence in taking the average amount of MSZW for each set of experiments in Table 3. Moreover, unlike for the decahydrate, the nucleation point for the monohydrate was repeatable.

**Figure 13:**
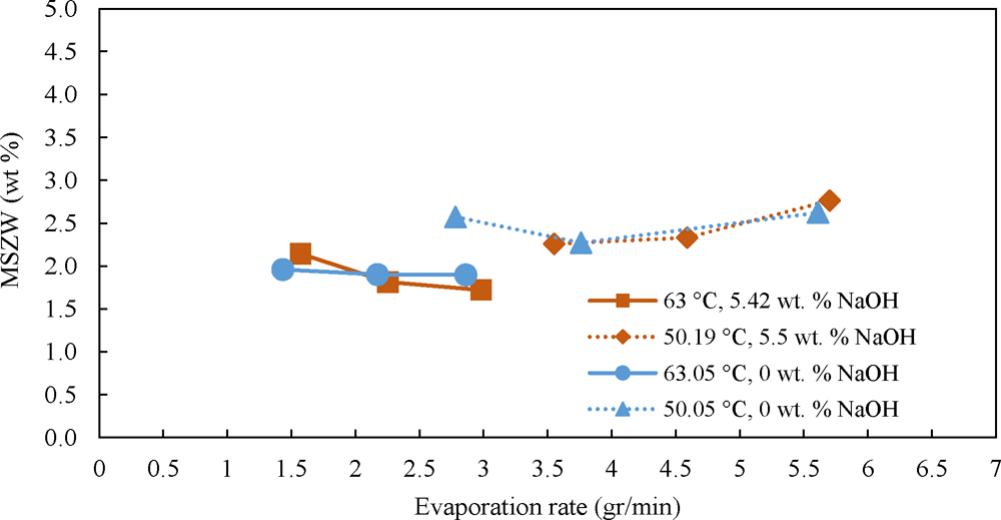
MSZW for Na_2_CO_3_·1H_2_O at different evaporation rates for temperatures of 50 and 63 °C, with and without NaOH.

**Figure 14:**
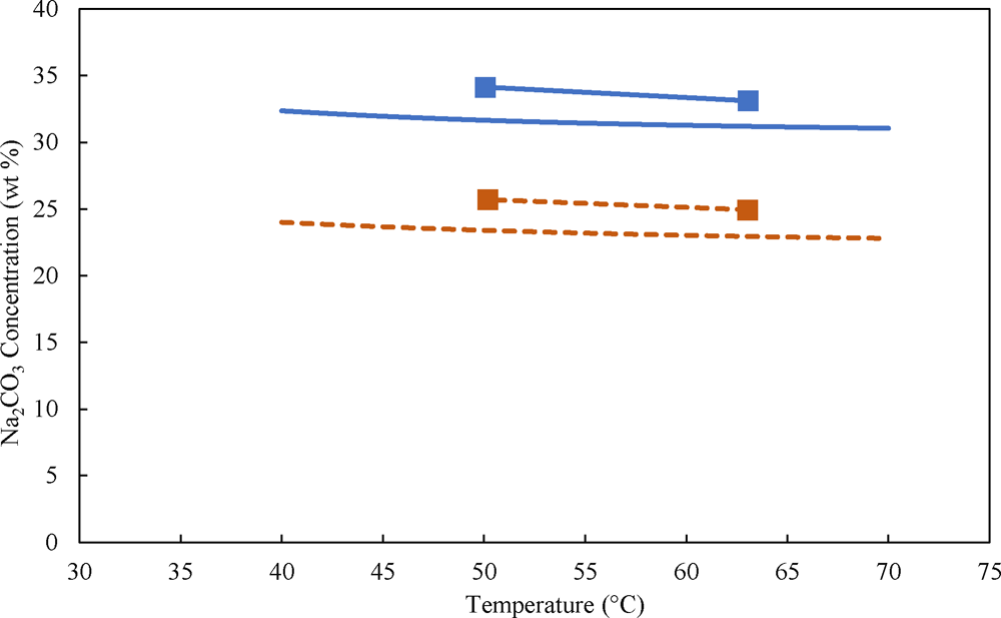
Solubility data obtained with the isothermal method from the polynomial fit without NaOH (blue solid line) and with 5.5 wt % NaOH (red dashed line). The obtained average MSZW of Na_2_CO_3_·1H_2_O in a 3L-scale at 50 and 63 °C, without NaOH (blue solid line with blue squares), and with 5.5 wt % NaOH (red dashed line with red squares). These MSZWs are the average of the MSZW obtained at different evaporation rates.

**Table 3:** Average Values of Repetition Experiments Conducted (as Referenced in S1.11) to Measure the MSZW of Na_2_CO_3_·1H_2_O, Using Vacuum Evaporative Crystallization at Constant Temperature and Constant Evaporation Rate

run no.	*T*_avg_ (°C)	*C*_NaOH, avg_ at nuc (wt %)	*C*_sat,avg_ at nuc (wt %)	*C*_Na2CO3,avg_ at nuc (wt %)	MSZW_avg_ (wt %)	*S*_avg_ at nuc
1.1–1.3	63.03	5.426	23.05	24.94	1.89	1.08
1.4–1.6	50.19	5.592	23.26	25.71	2.45	1.10
2.1–2.3	63.05	0	31.20	33.12	1.92	1.06
2.4–2.6	50.05	0	31.66	34.15	2.49	1.07

Since, the MSZW has to be considered for the seeding point and the seeding point should be closer to the solubility curve than to the supersolubility curve,^19^ the measured MSZW provides the necessary information to design appropriate experiments for future crystal growth kinetics studies. The seeding point needs to be within the MSZW, to prevent nucleation and at the same time take advantage of a high crystal growth rate. Additionally, the nucleated crystals at measured Δ*C*_max_ have regular shape and can be used as seeds with a controlled size by knowing the crystal growth rate, which is essential for initiating a continuous industrial-scale process.

## 4 Conclusions

In this work, the solid–liquid equilibria in the Na_2_CO_3_–NaOH-H_2_O system were studied under conditions relevant for crystallization of the Na_2_CO_3_ hydrates in combination with the CO_2_ DAC absorption process within the CODA project, a project dedicated to develop a novel carbon-negative strategy for soda ash production. For Na_2_CO_3_ decahydrate, 64 new SLE data points were obtained in the temperature range of 3.2–17.1 °C and NaOH concentrations ranging from 0 to 10 wt %. For the monohydrate, 36 new SLE data points cover the temperature range of 35–85 °C and NaOH concentrations of 0– 15 wt %. Literature data were selected for the conditions close to these conditions as well for each hydrate. The experimental data, along with the literature data, were used to correlate a polynomial fit with two variables for both the decahydrate and monohydrate. The study revealed that the solubility of decahydrate is highly dependent on temperature, whereas monohydrate exhibits slight retrograde solubility behavior. Additionally, the presence of NaOH reduces the solubility of both hydrates and influences the transition temperatures between them. The effect of NaOH concentration on transition between hydrates was studied at a constant temperature of 10 °C. The obtained polynomials were compared to the solubility determined with the eNRTL model. The findings indicate a discrepancy between the measured SLE data and the data predicted by the eNRTL model. This deviation is likely to become more pronounced during scale-up. Consequently, the derived polynomial fits exhibit greater reliability within the examined range of temperatures and NaOH concentrations. The MSZWs of Na_2_CO_3_·10H_2_O in the Na_2_CO_3_–NaOH-H_2_O system were reported for the first time in the temperature range of 29.6–9.3 °C both in 4 mL- and 3L-scale using cooling crystallization. The results revealed that the MSZW is stochastic and wider at higher than at lower temperatures, also the presence of NaOH extends this zone. From unfiltered solutions a decreasing trend of the MSZW is indicated; gas bubbles do not specifically affect it. In the bigger 3L-scale, the supersaturation ratio at nucleation point is in the range of 1.2–4.4. The MSZW for Na_2_CO_3_·1H_2_O was measured for the temperature range of 50–63 °C with different evaporation rates using vacuum evaporative crystallization at both constant temperature and evaporation rate for the first time as well. The obtained MSZW was shown to be almost independent of the evaporation rate and the presence of NaOH, and at 50 °C slightly wider than at 63 °C. With an average Δ*c*_max_ of 1.89–2.49 wt %, the MSZW was comparatively small and the supersaturation ratio at nucleation point about 1.1 within all the experiments performed. The obtained results give important insights for the operating conditions in the soda industry and also for designing proper experiments for studying the crystal growth kinetics in future work.

## Supplementary Material



## Notes

Detailed formulas used for the titration, details of thermodynamic model, experimental and literature SLE data, polynomial fits, and comprehensive information and results of the MSZW measurements (PDF)
