# Supplementary material for: Solubility and Metastable Zone Width Measurement of Na2CO3 Hydrate Phases in the Na2CO3–NaOH–H2O System as a Basis for a Novel Carbon-Negative Soda Ash Production Strategy
Source: Ind Eng Chem Res. 2025 May 12;64(21):10564–77. doi: 10.1021/acs.iecr.5c00320 (PMC12136036; doi:10.1021/acs.iecr.5c00320)
Supplement: Supplementary file 1 [file ie5c00320_si_002.pdf]

Supporting Information

Solubility and Metastable Zone Width Measurement of Na_2CO_3 Hydrate Phases in the Na_2CO_3-NaOH-H_2O System as a Basis for a Novel Carbon-Negative Soda Ash Production Strategy

Somayyeh Ghaffari *, Maria F. Gutierrez, Peter Schulze, Andreas Seidel-Morgenstern, Heike Lorenz

Max Planck Institute for Dynamics of Complex Technical Systems, Magdeburg, 39106 Germany

* ghaffari@mpi-magdeburg.mpg.de

1. Formulas for Eco-Titrator

The formulas to calculate the concentrations of NaOH and Na_2CO_3 according to the equivalence points obtained on Eco-Titrator are as follows:

$$C_{NaOH} = \frac{EP_1 \times C_{HCl} \times M_{NaOH}}{1000 \times W_{Analyte}} \times 100$$

$$C_{Na_2CO_3} = \frac{C_{HCl} \times 100 \times M_{Na_2CO_3}}{2 \times 2 \times 1000 \times W_{Analyte}} (2(EP_3 - EP_2) + (EP_3 - EP_2))$$

In these equations, C_{NaOH} represents the concentration of NaOH in wt. %, C_{HCl} denotes the concentration of acid in molarity, M_{NaOH} is the molecular weight of NaOH, while $W_{Analyte}$ is the weight of analyte in grams. $C_{Na_2CO_3}$ indicates the concentration of Na_2CO_3 in wt. %, Ep_1, Ep_2, and Ep_3 are the first, second, and third equivalence points, respectively, which refer to the consumed acid in milliliters for each equivalence point, and $M_{Na_2CO_3}$ is the molecular weight of Na_2CO_3.

2. Thermodynamic model of SLE

Calculation of the activity coefficient

$$\ln \gamma_i = \ln \gamma_i^{PDH} + \ln \gamma_i^{Born} + \ln \gamma_i^{lc}$$

$\ln \gamma_i^{PDH}$: Pitzer-Debye-Hückel contribution
$\ln \gamma_i^{Born}$: Born contribution
$\ln \gamma_i^{lc}$: local interaction contribution

The Pitzer-Debye-Hückel contribution is calculated as follows:

$$\ln \gamma_i^{PDH} = -\left(\frac{1000}{M_s}\right)^{\frac{1}{2}} A_\varphi \left[\left(\frac{2z_i}{\rho}\right) \ln\left(1 + \rho I_x^{1/2}\right) + \frac{z_i^2 I_x^{1/2} - 2I_x^{3/2}}{1 + \rho I_x^{1/2}}\right]$$

$$A_\varphi = \frac{1}{3}\left(\frac{2\pi N_A d_s}{1000}\right)^{1/2} \left(\frac{Q_e^2}{\varepsilon_s kT}\right)^{3/2}$$

$$I_x = \frac{1}{2}\sum_i x_i z_i^2$$

x_i: mole fraction of component i
M_s: molecular weight of the solvent (in this study pure water)
A_φ: Debye-Hückel parameter
N_A: Avogadro's number
d_s: mass density of solvent
Q_e: electron charge
ε_s: dielectric constant of the solvent
T: temperature
k: Boltzmann constant
I_x: ionic strength (mole fraction)
z_i: charge number of ion i
ρ: "closest approach" parameter

The Born contribution is calculated as follows:

$$\ln \gamma_i^{Born} = \frac{Q_e^2}{2kT}\left(\frac{1}{\varepsilon_s} - \frac{1}{\varepsilon_w}\right)\sum_i \frac{x_i z_i^2}{r_i} 10^{-2}$$

ε_w: dielectric constant of water
r_i: Born radius of the ionic species (assumed 3e-10 for all species)

In the case of pure water the $\ln \gamma_i^{Born}$ contribution is zero because $\varepsilon_s = \varepsilon_w$

Water dielectric constant

$$\varepsilon_w = 78.54 + 31989.38\left(\frac{1}{T} - \frac{1}{298.15}\right)$$

The local interaction contribution is calculated as follows (j and k can be any species anion, cation or molecule):

- For cations (subindex c):

$$\frac{1}{z_c}\ln \gamma_c^{lc} = \sum_c \sum_{a'} \frac{X_{a'}}{\sum_{a''} X_{a''} \sum_k X_k G_{kc,a'c}}\left(\tau_{Bc,a'c} - \frac{\sum_k X_k G_{kc,a'c}\tau_{kc,a'c}}{\sum_k X_k G_{kc,a'c}}\right) + \sum_B \frac{X_B G_{cB}}{\sum_k X_k G_{kB}}\left(\tau_{cB} - \frac{\sum_k X_k G_{kB}\tau_{kB}}{\sum_k X_k G_{kB}}\right) + \sum_a \sum_{c'} \frac{X_{c'}}{\sum_{c''} X_{c''} \sum_k X_k G_{ka,c'a}}\left(\tau_{ca,c'a} - \frac{\sum_k X_k G_{ka,c'a}\tau_{ka,c'a}}{\sum_k X_k G_{ka,c'a}}\right)$$

- For anions (subindex a):

$$\frac{1}{z_a}\ln \gamma_a^{lc} = \sum_a \sum_{c'} \frac{X_{c'}}{\sum_{c''} X_{c''} \sum_k X_k G_{ka,c'a}}\left(\tau_{aB} - \frac{\sum_k X_k G_{kB}\tau_{kB}}{\sum_k X_k G_{kB}}\right) + \sum_B \frac{X_B G_{aB}}{\sum_k X_k G_{kB}}\left(\tau_{aB} - \frac{\sum_k X_k G_{kB}\tau_{kB}}{\sum_k X_k G_{kB}}\right) + \sum_c \sum_{a'} \frac{X_{a'}}{\sum_{a''} X_{a''} \sum_k X_k G_{kc,a'c}}\left(\tau_{ac,a'c} - \frac{\sum_k X_k G_{kc,a'c}\tau_{kc,a'c}}{\sum_k X_k G_{kc,a'c}}\right)$$

- For molecules (subindex B):

$$\ln \gamma_B^{lc} = \frac{\sum_j X_j G_{jB} \tau_{jB}}{\sum_k X_k G_{kB}} + \sum_{B'} \frac{X_{B'} G_{BB'}}{\sum_k X_k G_{kB'}} \left(\tau_{BB'} - \frac{\sum_k X_k G_{kB'} \tau_{kB'}}{\sum_k X_k G_{kB'}} \right)$$
$$+ \sum_c \sum_{a'} \frac{X_{a'}}{\sum_{a''} X_{a''} \sum_k X_k G_{kc,a'c}} \left(\tau_{Bc,a'c} - \frac{\sum_k X_k G_{kc,a'c} \tau_{kc,a'c}}{\sum_k X_k G_{kc,a'c}} \right)$$
$$+ \sum_a \sum_{c'} \frac{X_{c'}}{\sum_{c''} X_{c''} \sum_k X_k G_{ka,c'a}} \left(\tau_{Ba,c'a} - \frac{\sum_k X_k G_{ka,c'a} \tau_{ka,c'a}}{\sum_k X_k G_{ka,c'a}} \right)$$

In the previous equations:

$$G_{cB} = \frac{\sum_a X_a G_{ca,B}}{\sum_{a'} X_{a'}}$$

$$G_{aB} = \frac{\sum_c X_c G_{ca,B}}{\sum_{c'} X_{c'}}$$

$$\alpha_{Bc} = \alpha_{cB} = \frac{\sum_a X_a \alpha_{Bc,a}}{\sum_{a'} X_{a'}}$$

$$\alpha_{Ba} = \alpha_{aB} = \frac{\sum_c X_c \alpha_{Ba,c}}{\sum_{c'} X_{c'}}$$

$$\tau_{cB} = -\frac{\ln G_{cB}}{\alpha_{cB}}$$

$$\tau_{aB} = -\frac{\ln G_{aB}}{\alpha_{aB}}$$

$$\tau_{Ba,ca} = \tau_{aB} - \tau_{ca,B} + \tau_{B,ca}$$

$$\tau_{Bc,ac} = \tau_{cB} - \tau_{ca,B} + \tau_{B,ca}$$

$$G_{ca,B} = \exp(-\alpha_{ca,B} \tau_{ca,B})$$
$$G_{ca,ca'} = \exp(-\alpha_{ca,ca'} \tau_{ca,ca'})$$

The equations to obtain the interaction parameters are:

Electrolyte (ca)-Molecule (B) pair parameters

$$\tau_{B,ca} = C_{B,ca} + \frac{D_{B,ca}}{T} + E_{B,ca} \left(\frac{T^{ref} - T}{T} + \ln \left(\frac{T}{T^{ref}} \right) \right)$$

$$\tau_{ca,B} = C_{ca,B} + \frac{D_{ca,B}}{T} + E_{ca,B} \left(\frac{T^{ref} - T}{T} + \ln \left(\frac{T}{T^{ref}} \right) \right)$$

Electrolyte (ca)-Electrolyte (ca') pair parameters

$$\tau_{ca,ca'} = C_{ca,ca'} + \frac{D_{ca,ca'}}{T} + E_{ca,ca'}\left(\frac{T^{ref} - T}{T} + \ln\left(\frac{T}{T^{ref}}\right)\right)$$

$$\tau_{ca',ca} = C_{ca',ca} + \frac{D_{ca',ca}}{T} + E_{ca',ca}\left(\frac{T^{ref} - T}{T} + \ln\left(\frac{T}{T^{ref}}\right)\right)$$

Table S1. $C_{B,ca}$,$C_{ca,B}$, $C_{ca,ca'}$, $C_{ca',ca}$ parameters used in the eNRTL model

		$\boldsymbol{B}$	$\boldsymbol{ca}$	$\boldsymbol{ca'}$
		$\boldsymbol{H_2O}$	$\boldsymbol{Na^+OH^-}$	$\boldsymbol{Na^+CO_3^{2-}}$
$\boldsymbol{B}$	$\boldsymbol{H_2O}$	0	6.737997	-4.833
$\boldsymbol{ca}$	$\boldsymbol{Na^+OH^-}$	-3.771221	0	0
$\boldsymbol{ca'}$	$\boldsymbol{Na^+CO_3^{2-}}$	0.977	0	0

Table S2. $D_{B,ca}$, $D_{ca,B}$, $D_{ca,ca'}$, $D_{ca',ca}$ parameters used in the eNRTL model

		$\boldsymbol{B}$	$\boldsymbol{ca}$	$\boldsymbol{ca'}$
		$\boldsymbol{H_2O}$	$\boldsymbol{Na^+OH^-}$	$\boldsymbol{Na^+CO_3^{2-}}$
$\boldsymbol{B}$	$\boldsymbol{H_2O}$	0	1420.242	4018.4
$\boldsymbol{ca}$	$\boldsymbol{Na^+OH^-}$	-471.8202	0	0
$\boldsymbol{ca'}$	$\boldsymbol{Na^+CO_3^{2-}}$	-1547	0	0

Table S3. $E_{B,ca}$, $E_{ca,B}$, $E_{ca,ca'}$, $E_{ca',ca}$ parameters used in the eNRTL model

		$\boldsymbol{B}$	$\boldsymbol{ca}$	$\boldsymbol{ca'}$
		$\boldsymbol{H_2O}$	$\boldsymbol{Na^+OH^-}$	$\boldsymbol{Na^+CO_3^{2-}}$
$\boldsymbol{B}$	$\boldsymbol{H_2O}$	0	3.013932	88.56
$\boldsymbol{ca}$	$\boldsymbol{Na^+OH^-}$	2.136557	0	0
$\boldsymbol{ca'}$	$\boldsymbol{Na^+CO_3^{2-}}$	-32.4	0	0

Table S4. $\alpha_{B,ca}$, $\alpha_{ca,B}$, $\alpha_{ca,ca'}$, $\alpha_{ca',ca}$ parameters used in the eNRTL model

		$\boldsymbol{B}$	$\boldsymbol{ca}$	$\boldsymbol{ca'}$
$\boldsymbol{B}$	$\boldsymbol{H_2O}$	$\boldsymbol{H_2O}$	$\boldsymbol{Na^+OH^-}$	$\boldsymbol{Na^+CO_3^{2-}}$
$\boldsymbol{ca}$	$\boldsymbol{Na^+OH^-}$	0	0.2	0.2
$\boldsymbol{ca'}$	$\boldsymbol{Na^+CO_3^{2-}}$	0.2	0	0.2
$\boldsymbol{B}$	$\boldsymbol{H_2O}$	0.2	0.2	0

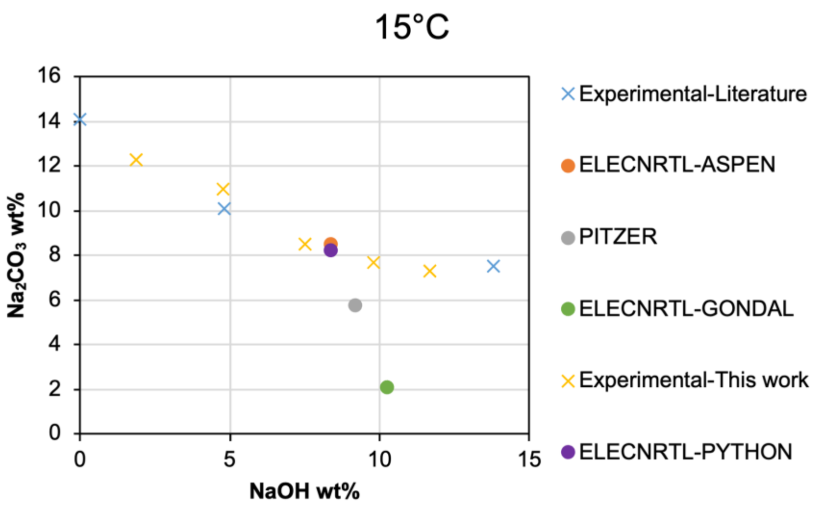

Figure S1. Validation of the activity coefficient and solubility calculations.

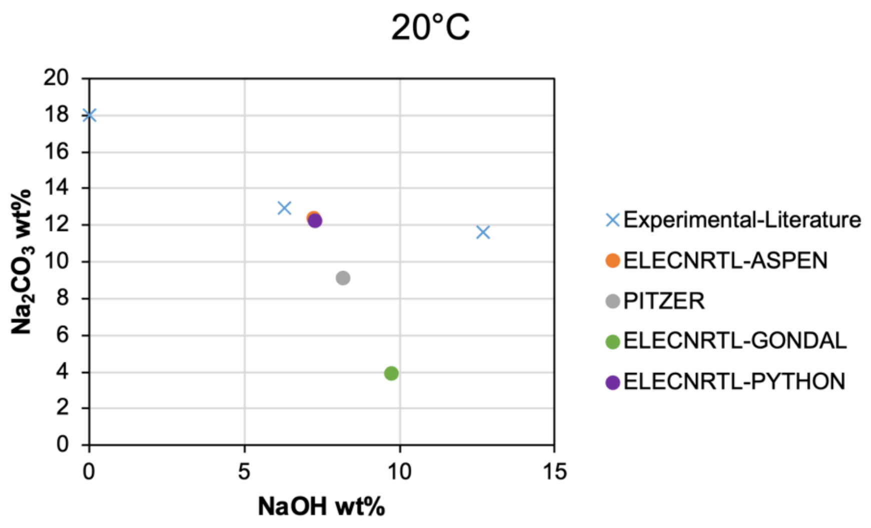

Figure S2. Validation of the activity coefficient and solubility calculations.

3. SLE of the $Na_2CO_3{\cdot}H_2O$, NaOH, and H_2O system

3.1. Effect of NaOH

Table S5. SLE data of Na_2CO_3 at a temperature around 10°C, and different concentrations of NaOH together with the obtained solid phase.

Name	T (°C)	NaOH (wt. %)	Na_2CO_3 (wt. %)	Solid phase
1	9.9	0	11.393	Deca
2	10	1.63	9.99	Deca
3	10	3.65	8.47	Deca
4	9.9	3.86	7.65	Deca
5	9.9	5.41	6.64	Deca
6	9.9	7.88	5.91	Deca
7	9.9	8.75	6.06	Deca
8	9.9	10.09	5.09	Deca
10	10	12.89	4.54	Deca
11	10	14.69	4.86	Deca
12	9.9	15.44	5.12	Deca

13	9.9	19.04	6.38	Hepta
14	9.9	30.47	0.82	Mono

3.2.Literature SLE data of $Na_2CO_3 \cdot 10H_2O$

Table S6. Literature SLE data of $Na_2CO_3 \cdot 10H_2O$ in the temperature range of 5-30 °C , and a NaOH concentration range of 0-12.7 wt. %.

Sample number	T (°C)	C_{NaOH} (wt. %)	C_{Na2CO3} (wt. %)	Ref.	Sample number	T (°C)	C_{NaOH} (wt. %)	C_{Na2CO3} (wt. %)	Ref.
**1**	5	3.51	6.06	[7]	14	25	0	22.7	[14]
**2**	5	11.6	3.11	[7]	15	25	2.09	20.54	[7]
**3**	5	12.08	3.24	[7]	16	25	5.4	18.2	[14]
**4**	10	2.1	9.18	[7]	17	25	7	18.1	[10, 14]
**5**	10	9.02	5.51	[7]	18	25	7.42	17.5	[7]
**6**	15	0	14.1	[10]	19	30	0	28.4	[10]
**7**	15	4.8	10.1	[10]	20	30	0	29.04	[15, 16]
**8**	15	13.8	7.5	[10]	21	30	1.55	27.23	[16]
**9**	20	0	18	[10]	22	30	1.75	27.03	[16]
**10**	20	6.3	12.9	[10]	23	30	1.97	27.18	[16]
**11**	20	10.04	11.48	[7]	24	30	2.1	27.16	[16]
**12**	20	11.01	11.59	[7]	25	30	2.94	26.78	[16]
**13**	20	12.7	11.6	[10]	26	30	3.1	26.5	[15]

3.3.Literature SLE data of $Na_2CO_3 \cdot 1H_2O$

Table S7. Literature SLE data of $Na_2CO_3 \cdot 1H_2O$ in the temperature range of 30-100°C, and a NaOH concentration range of 0-16.86 wt. %.

Sample number	T (°C)	C_{NaOH} (wt. %)	C_{Na2CO3} (wt. %)	Ref.	Sample number	T (°C)	C_{NaOH} (wt. %)	C_{Na2CO3} (wt. %)	Ref.
**1**	30	9.34	19.7	[16]	19	70	3.9	25.2	[7]
**2**	30	11.26	16.69	[16]	20	70	7.94	19.88	[7]
**3**	30	14.1	13.39	[16]	21	70	16.2	10.14	[7]
**4**	35	3.02	28.2	[7]	22	80	0	31	[9]
**5**	35	4.9	25.3	[14]	23	80	3.98	25.01	[7]
**6**	35	7.19	20.71	[7]	24	80	6.04	22	[7]
**7**	35	11.9	15.2	[14]	25	80	14.1	12.27	[9]
**8**	40	5.23	24.63	[7]	26	80	16.86	9.58	[9]
**9**	50	0	32.19	[13]	27	90	6.34	21.2	[7]
**10**	50	4.21	26.01	[7]	28	100	0	30.9	[9]
**11**	50	8.84	18.39	[11]	29	100	4.29	25.1	[9]
**12**	50	14.2	11.92	[7]	30	100	8.09	19.96	[12]
**13**	60	0	31.85	[9]	31	100	10.51	17.32	[9]
**14**	60	1.96	28.02	[7]	32	100	10.96	16.27	[7]
**15**	60	5.92	23.39	[9]	33	100	12.18	15.08	[12]
**16**	60	7.22	20.99	[7]	34	100	12.5	14.26	[12]
**17**	60	15.4	10.95	[9]	35	100	12.86	14.45	[9]
**18**	60	15.8	10.61	[7]	36	100	13.08	13.65	[12]

3.4 Polynomial fits

3.4.1 Polynomial fit for $Na_2CO_3 \cdot 10H_2O$

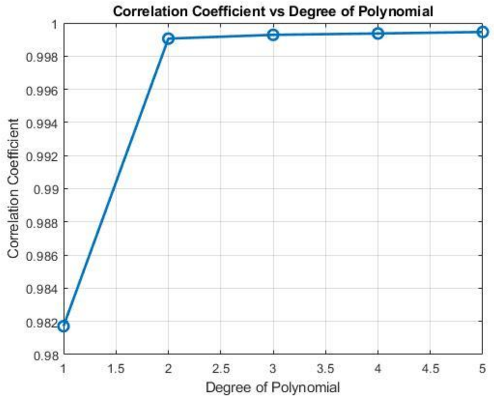

Figure S3. Correlation coefficient for various degree of polynomial for SLE data of $Na_2CO_3 \cdot 10H_2O$

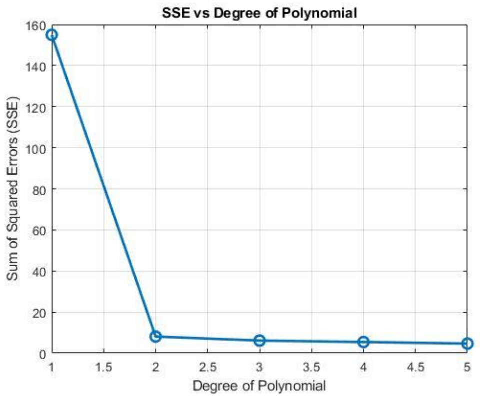

Figure S4. SSE for various degree of polynomial for SLE data of $Na_2CO_3 \cdot 10H_2O$

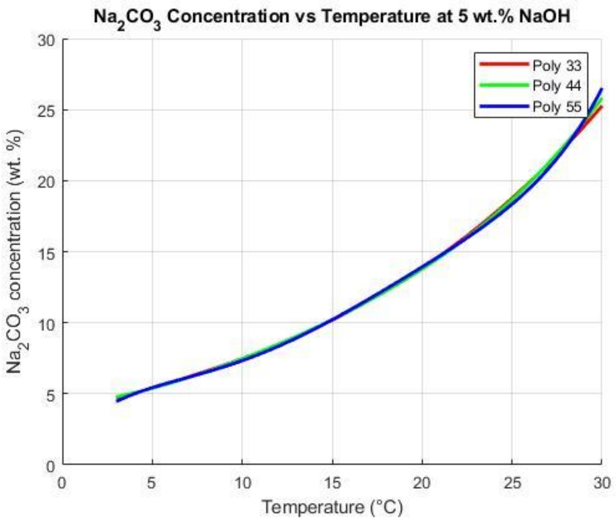

Figure S5. $Na_2CO_3 \cdot 10H_2O$ solubility calculated with various polynomial degree

Equation for the polynomial fit of the decahydrate, derived using both experimental and literature data

$$w_{Na_2CO_3} = p_{00} + p_{10}\,T + p_{01}\,w_{NaOH} + p_{20}\,T^2 + p_{11}\,T\,w_{NaOH} + p_{02}\,w_{NaOH}^2 + p_{30}\,T^3 + p_{21}\,T^2\,w_{NaOH} + p_{12}\,T\,w_{NaOH}^2 + p_{03}\,w_{NaOH}^3 + p_{40}\,T^4 + p_{31}\,T^3\,w_{NaOH} + p_{22}\,T^2\,w_{NaOH}^2 + p_{13}\,T\,w_{NaOH}^3 + p_{04}\,w_{NaOH}^4 + p_{50}\,T^5 + p_{41}\,T^4\,w_{NaOH} + p_{32}\,T^3\,w_{NaOH}^2 + p_{23}\,T^2\,w_{NaOH}^3 + p_{14}\,T\,w_{NaOH}^4 + p_{05}\,w_{NaOH}^5$$

Units: $w_{Na_2CO_3}$ $in\ wt.\%$, $T\ in\ °C$ and w_{NaOH} $in\ wt.\%$

Table S8. Coefficient values and the 95% confidence interval bounds values of the polynomial fit for $Na_2CO_3{\cdot}10H_2O$ using experimental and literature data.

Coefficient	Value	95% confidence interval bounds	
p_{00}	3.092	-0.4727	6.656
p_{10}	1.988	0.5376	3.439
p_{01}	-0.3143	-1.627	0.998
p_{20}	-0.2613	-0.475	-0.04756
p_{11}	-0.1167	-0.4111	0.1778
p_{02}	-0.1231	-0.5832	0.337
p_{30}	0.02021	0.005788	0.03463
p_{21}	0.01214	-0.01618	0.04046
p_{12}	0.002638	-0.03813	0.04341
p_{03}	0.04196	-0.03896	0.1229
p_{40}	-0.00068	-0.001129	-0.0002279
p_{31}	-0.00057	-0.001695	0.0005528
p_{22}	-0.00044	-0.002705	0.001834
p_{13}	-2.47E-05	-0.003171	0.003121
p_{04}	-0.00415	-0.01035	0.002043
p_{50}	8.45E-06	3.162e-06	1.374e-05
p_{41}	9.13E-06	-6.484e-06	2.475e-05
p_{32}	1.52E-05	-2.667e-05	5.707e-05
p_{23}	-2.29E-06	-5.97e-05	5.512e-05
p_{14}	1.30E-05	-9.743e-05	0.0001234
p_{05}	0.000129	-4.883e-05	0.0003066

3.4.2 Polynomial fit for $Na_2CO_3{\cdot}$ $1H_2O$

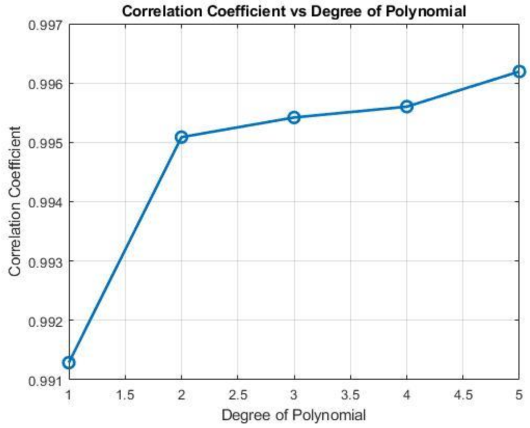

Figure S6. Correlation coefficient for various degree of polynomial for SLE data of $Na_2CO_3{\cdot}$ $1H_2O$

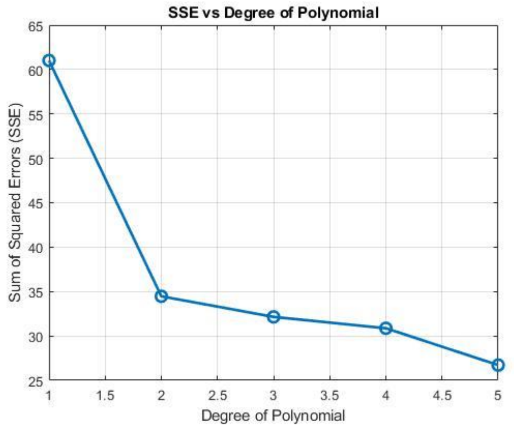

Figure S7. SSE for various degree of polynomial for SLE data of $Na_2CO_3 \cdot 1H_2O$

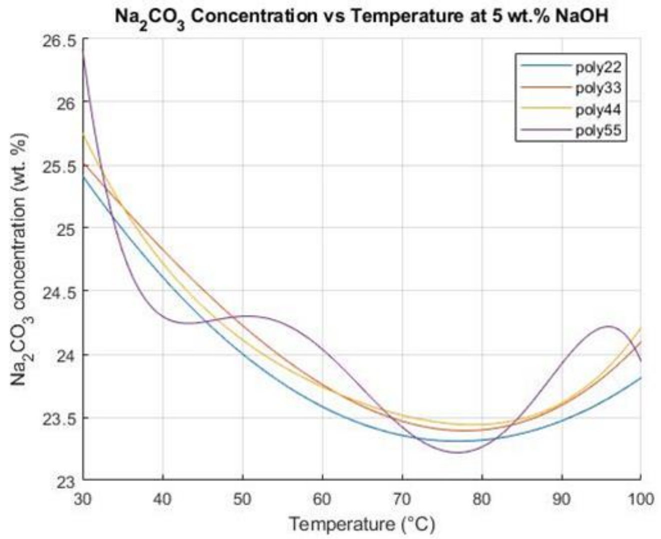

Figure S8. Solubility data of $Na_2CO_3 \cdot 1H_2O$ calculated with various polynomial degree

Equation for the polynomial fit of the monohydrate, derived using both experimental and literature data$w_{Na_2CO_3} = p_{00} + p_{10}\,T + p_{01}\,w_{NaOH} + p_{20}\,T^2 + p_{11}\,T\,w_{NaOH} + p_{02}\,w_{NaOH}^2 + p_{30}\,T^3 + p_{21}\,T^2\,w_{NaOH} + p_{12}\,T\,w_{NaOH}^2 + p_{03}\,w_{NaOH}^3 + p_{40}\,T^4 + p_{31}\,T^3\,w_{NaOH} + p_{22}\,T^2\,w_{NaOH}^2 + p_{13}\,T\,w_{NaOH}^3 + p_{04}\,w_{NaOH}^4$

Units: $w_{Na_2CO_3}$ $in\ wt.\%$, T $in\ °C$ and w_{NaOH} $in\ wt.\%$

Table S9. Coefficient values and the 95% confidence interval bounds values of the polynomial fit for $Na_2CO_3 \cdot 1H_2O$ using experimental and literature data.

Coefficient	Value	95% confidence interval bounds	
p_{00}	45.12	17.81	72.43
p_{10}	-0.6736	-2.421	1.074
p_{01}	-2.33	-4.813	0.1537
p_{20}	0.01276	-0.02801	0.05352
p_{11}	0.03128	-0.05709	0.1197
p_{02}	0.06918	-0.1674	0.3058
p_{30}	-0.00011	-0.0005237	0.0002987
p_{21}	-0.00049	-0.001598	0.0006131
p_{12}	-0.0003	-0.004349	0.003741

**p_{03}**	-0.00641	-0.02174	0.008926
**p_{40}**	3.81E-07	-1.134e-06	1.897e-06
**p_{31}**	2.76E-06	-2.164e-06	7.689e-06
**p_{22}**	-2.41E-06	-2.455e-05	1.974e-05
**p_{13}**	3.15E-05	-7.858e-05	0.0001416
**p_{04}**	0.000196	-0.0002835	0.0006744

3.4.3 Polynomial fit for Na_2CO_3

Equation for the polynomial fit of the anhydrous Na_2CO_3, derived using only literature data

$$w_{Na_2CO_3} = p_{00} + p_{10}\,T + p_{01}\,w_{NaOH} + p_{20}\,T^2 + p_{11}\,T\,w_{NaOH} + p_{02}\,w_{NaOH}^2 + p_{30}\,T^3 + p_{21}\,T^2\,w_{NaOH} + p_{12}\,T\,w_{NaOH}^2 + p_{03}\,w_{NaOH}^3 + p_{40}\,T^4 + p_{31}\,T^3\,w_{NaOH} + p_{22}\,T^2\,w_{NaOH}^2 + p_{13}\,T\,w_{NaOH}^3 + p_{04}\,w_{NaOH}^4 + p_{50}\,T^5 + p_{41}\,T^4\,w_{NaOH} + p_{32}\,T^3\,w_{NaOH}^2 + p_{23}\,T^2\,w_{NaOH}^3 + p_{14}\,T\,w_{NaOH}^4 + p_{05}\,w_{NaOH}^5$$

Units: $w_{Na_2CO_3}$ $in\ wt.\%$, $T\ in\ °C$ and w_{NaOH} $in\ wt.\%$

Table S10. Coefficient values and the 95% confidence interval bounds values of the polynomial fit for Na_2CO_3 using only literature data.

Coefficient	Value	95% confidence interval bounds	
p_{00}	-1.685	0.15991985	1.36390119
p_{10}	0.03747	-0.0706447	0.01261906
p_{01}	12.24	-12.895047	0.87645935
p_{20}	-0.000166	-0.000375	0.00185921
p_{11}	-0.2433	-0.1120173	0.61888781
p_{02}	-35.13	-15.600032	52.7166055
p_{30}	-3.24E-07	-2.38E-05	5.44E-06
p_{21}	0.001195	-0.0122516	0.00188128
p_{12}	0.5188	-2.0684486	0.49727717
p_{03}	55.75	-128.73271	56.8088163
p_{40}	1.54E-09	-3.97E-08	1.48E-07
p_{31}	-4.94E-08	-1.48E-05	0.00010347
p_{22}	-0.002356	-0.0045064	0.02702538
p_{13}	-0.411	-1.0815385	3.19962923
p_{04}	-55.17	-78.849945	194.405185
p_{50}	5.07E-12	-3.56E-10	1.17E-10

4. MSZWs measurements details

Table S11. Details of the experiments (saturation concentration (C_{Na2CO3}), saturation temperature by isothermal method (T_{eq}), cooling rate (CR), nucleation temperature (T_{nuc}), MSZW ($\Delta Tmax$) used for detecting the nucleation points of $Na_2CO_3{\cdot}10H_2O$ with and without NaOH via Crystalline®. Solutions were filtered to remove the particles bigger than 0.2 µm. Details of the experiments for measuring the solubility (equilibrium) temperature with the polythermal method. (heating rate(HR), dissolution temperature (T_{diss}), equilibrium temperature by polythermal method(T_{eq}**))*

Run No.	C_{Na2CO3} (wt.%)	C_{NaOH} (wt.%)	T_{eq}* (°C)	CR (K/h)	T_{nuc} (°C)	ΔT_{max} (K)	HR (K/h)	T_{diss} (°C)	T_{eq}** (°C)
1.1	26.31	0	28.39	6	-2.7	31.09	6	30.03	28.22
1.2				12	-3.4	31.79	12	31.9	
1.3				18	-2.9	31.29	18	33.2	
1.4				24	-3.5	31.89	24	39.3	
2.1	21.68	0	23.88	12	2.1	21.78	6	25.6	24.56
2.2				18	3.6	20.28	12	26.9	
2.3				24	8.4	15.48	18	27.8	

3.1	14.17	0	14.71	6	1.3	13.41	6	16.0	15.1
3.2				12	1	13.71	12	16.5	
3.3				18	-2.3	17.01	18	17.6	
3.4				24	-5.5	20.21	-	-	
4.1	11.11	0	10	6	-3.4	13.4	6	14.1	12.04
4.2				12	-2.3	12.3	12	24.5	
4.3				18	-4.9	14.9	18	26.0	
4.4				24	-5.4	15.4	24	27.9	
5.1	21.86	4.87	27.67	6	0.1	27.57	6	29.40	27.01
5.2				12	-1.3	28.97	12	30.30	
5.3				18	-1.1	28.77	18	31.60	
5.4				24	-3.3	30.97	36	37.70	
6.1	16.03	4.96	22.55	6	-5.7	28.25	6	25.3	22.43
6.2				12	-10.7	33.25	12	24.3	
6.3				18	-10.1	32.65	18	25.3	
6.4				24	-7.7	30.25	-	-	
7.1	7	4.89	9.12	6	-9.7	18.82	6	10.7	10.23
7.2				12	-11.1	20.22	12	11.3	
7.3				18	-13.5	22.62	18	11.7	
7.4				24	-14.6	23.72	-	-	

*refers to solubility temperature calculated with polynoms obtained for decahydrate
**refers to solubility temperature obtained by polythermal method (extrapolating dissolution temperature to the lowest heating rate)

Table S12. Details of the experiments (saturation concentration (C_{Na2CO3}), saturation temperature (Teq), cooling rate (CR), nucleation temperature (T_{nuc}), MSZW (ΔT_{max}) used for detecting the nucleation points of $Na_2CO_3{\cdot}10H_2O$ with and without NaOH via Crystalline®. Solutions were not filtered to remove small particles.

Run No.	C_{Na2CO3} (wt. %)	C_{NaOH} (wt. %)	T_{eq} (°C)	ΔT_{max} (K)	CR (K/h)	T_{nuc} (°C)
1.1	26.31	0	28.39	23.39	12	5
1.2				25.89	18	2.5
1.3				23.49	24	4.9
2.1	21.68	0	23.88	15.48	12	8.4
2.2				20.28	18	3.6
2.3				21.78	24	2.1
3.1	14.17	0	14.71	16.81	12	-2.1
3.2				21.01	18	-6.3
3.3				21.11	24	-6.4
4.1	11.11	0	10	13.6	12	-3.6
4.2				10.5	18	-0.5
4.3				13.2	24	-3.2
5.1	21.86	4.87	27.67	31.77	12	-4.1
5.2				21.47	18	6.2
5.3				18.37	24	9.3
6.1	16.03	4.96	22.55	30.45	12	-7.9
6.2				30.65	18	-8.1
6.3				26.85	24	-4.3
7.1	7	4.89	9.12	15.72	12	-6.6
7.2				14.42	18	-5.3
7.3				15.82	24	-6.7

Table S13. Details of the experiments (saturation concentration (C_{Na2CO3}), saturation temperature by isothermal method (Teq), cooling rate (CR), nucleation temperature (T_{nuc}), MSZW (ΔT_{max}), saturation concentration at nucleation temperature, supersaturation ratio at the nucleation point (S) used for detecting the nucleation points of $Na_2CO_3{\cdot}10H_2O$ with and without*

*NaOH in a 3L-scale set-up. Details of the experiments for measuring solubility (equilibrium) temperature based on polythermal method (heating rate (HR), dissolution temperature (T_{diss}), equilibrium temperature by polythermal method (Teq**)).*

Run No.	C_{Na2CO3} (wt. %)	C_{NaOH} (wt.%)	T_{eq}^{*} (°C)	CR (K/h)	T_{nuc} (°C)	ΔT_{max} (K)	c_{sat} at T_{nuc} (wt. %)	S at nuc	HR (K/h)	T_{diss} (°C)	T_{eq}^{***} (°C)
1.1	28.11	0	29.6	5.94	19.39	10.21	17.95	1.57	-	-	-
1.2	28.11	0	29.6	10.9	15.23	14.37	14.56	1.93	-	-	
1.3	28.11	0	29.6	11.98	15	14.6	14.39	1.95			
1.4	28.11	0	29.6	30	15.68	13.92	14.91	1.89	-	-	
2.1	20.94	0	23	5.65	12.38	10.62	12.52	1.67	-	-	-
2.2	20.94	0	23	11.98	7.29	15.71	9.78	2.14	-	-	
2.3	20.94	0	23	30	6	17	9.16	2.29			
2.4	20.94	0	23	32.36	7.27	15.73	9.78	2.14	-	-	
3.1	14.19	0	14.74	5.94	9.32	5.42	10.76	1.32	11.88	15.56	14.57
3.2	14.19	0	14.74	11.29	11.64	3.1	12.05	1.18			14.57
3.3	14.19	0	14.74	11.99	8.51	6.23	10.36	1.37	29.71	17.04	14.57
3.4	14.19	0	14.74	34.97	7.88	6.86	10.06	1.41	-	-	14.57
3.5	14.19	0	14.74	29.98	4.82	9.92	8.52	1.67	-	-	14.57
4.1	10.79	0	9.38	5.94	3	6.38	7.2	1.50			10.17
4.2	10.79	0	9.38	6.74	0.95	8.43	NA	NA	11.22	9.99	10.17
4.3	10.79	0	9.38	11.98	4	5.38	7.99	1.35			10.17
4.4	10.79	0	9.38	14.95	5.97	3.41	9.15	1.18	18.36	9.87	10.17
4.5	10.79	0	9.38	22.57	-4.14	13.52	NA	NA	-	-	10.17
4.6	10.79	0	9.38	30	2	7.38	NA	NA			10.17
5.1	22.33	4.96	27.97	5.94	8	19.97	6.53	3.42			27.26
5.2	22.33	4.96	27.97	6.31	8.68	19.29	6.79	3.29	36.60	27.65	27.26
5.3	22.33	4.96	27.97	11.98	8.76	19.21	6.82	3.27	22.34	27.79	27.26
5.4	22.33	4.96	27.97	30	9.5	18.47	7.12	3.14			27.26
5.5	22.33	4.96	27.97	34.66	10.82	17.15	7.79	2.87	4.18	27.23	27.26
6.1	16.43	4.85	22.94	5.99	7.55	15.39	6.41	2.56	6.67	23.19	23.38
6.2	16.43	4.85	22.94	11.98	5.46	17.48	5.66	2.90	9.43	22.87	23.38
6.3	16.43	4.85	22.94	23.21	7.1	15.84	6.25	2.63	6.57	23.16	23.38
6.4	16.43	4.85	22.94	24.46	2.01	20.93	3.77	4.36	-	-	23.38
6.5	16.43	4.85	22.94	30	2.5	20.44	4.07	4.04			23.38
7.1	6.96	5.17	9.35	5.94	6.33	3.02	5.85	1.19	5.98	9.94	9.67
7.2	6.96	5.17	9.35	11.98	0.98	8.37	2.73	2.55			9.67
7.3	6.96	5.17	9.35	12.65	1.55	7.8	3.31	2.10	15.38	9.62	9.67
7.4	6.96	5.17	9.35	23.32	5.34	4.01	5.5	1.27	28.76	10.24	9.67
7.5	6.96	5.17	9.35	30	4	5.35	5.02	1.39	-		9.67

*refers to solubility temperature calculated with polynoms obtained for each hydrate using data obtained with excess method
***refers to solubility temperature with polythermal method.

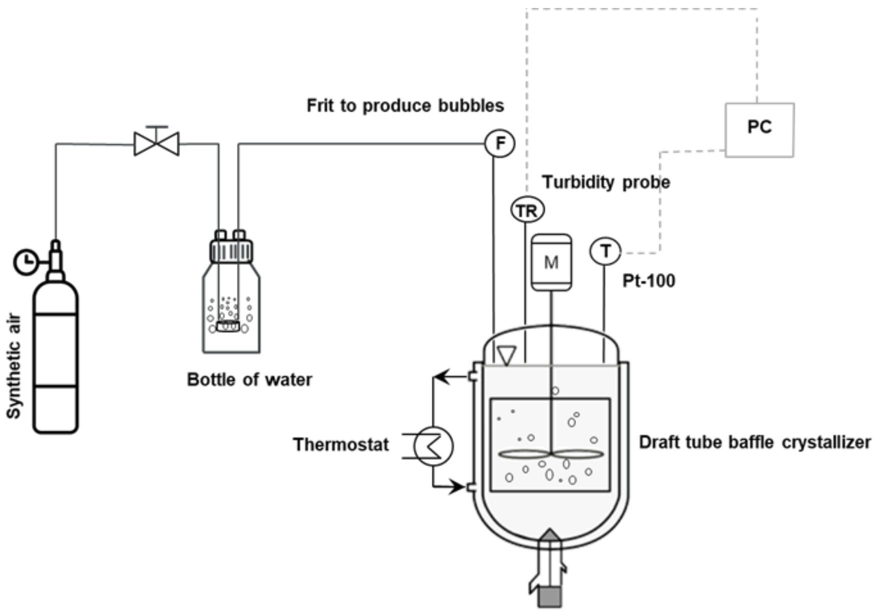

Figure S9. Flow chart of the used experimental set-up for measuring the effect of bubbles on MSZW.

Table S14. Details of the experiments (solution concentration (C_{Na2CO3}), saturation temperature (T_{eq}), cooling rate (CR), nucleation temperature (T_{nuc}), MSZW (ΔT_{max}), used for detecting the nucleation points of $Na_2CO_3 \cdot 10H_2O$ with NaOH in a 3L-scale set-up with bubbling.

Run No.	C_{Na2CO3} (wt.%)	C_{NaOH} (wt.%)	T_{eq} (°C)	CR (K/h)	T_{nuc} (°C)	ΔT_{max} (K)
8.1	16.82	4.94	23.45	7.8	6.33	17.12
8.2				12	10.29	13.16
8.3				24	5.93	17.52
9.1	7.05	5.08	9.58	6	2.24	7.01
9.2				12	0.16	9.42
9.3				13.8	-10.14	19.72
9.4				20.4	-5.24	14.82

*Table S15. Details of the experiments (solution concentration (C_{Na2CO3}), temperature applied on thermostat (T_{tmo}), evaporation rate (ER), controlled average temperature during evaporation (T_{avg}), applied initial vacuum pressure (P_{int}), amount of solution, NaOH concentration at nucleation point, saturation concentration of Na_2CO_3 at nucleation point, MSZW (**ΔC_{max}**), and super-saturation ratio at the nucleation point (S)) used for measuring the MSZW of $Na_2CO_3 \cdot 1H_2O$ with and without NaOH in a 3L-scale set-up.*

Run No.	C_{Na2CO3} (wt. %)	C_{NaOH} (wt. %)	T_{tmo} (°C)	ER (gr/min)	T_{avg} (°C)	$P_{init.}$ (mbar)	Solution amount (gr)	C_{NaOH} at nuc (wt.%)	c_{sat} at T_{nuc} (wt.%)	C_{Na2CO3} at nuc (wt. %)	ΔC_{max} (wt. %)	S_{MSZW} -
**1.1**	22.63	4.92	70	1.57	63.06	180	3095.54	5.47	22.99	25.13	2.14	1.09
**1.2**	22.48	4.89	72	2.25	63.03	177	3096.24	5.41	23.07	24.89	1.82	1.08
**1.3**	22.59	4.91	74	2.98	63	177	3101.5	5.4	23.09	24.81	1.72	1.07
**1.4**	22.48	4.89	62	3.55	50.31	94	3116.9	5.56	23.30	25.56	2.26	1.10
**1.5**	22.54	4.9	65	4.59	50.26	95	3109.2	5.57	23.29	25.62	2.33	1.10
**1.6**	22.63	4.92	68	5.7	50	94	3096.24	5.65	23.19	25.95	2.76	1.12
**2.1**	30.95	0	70	1.43	63.06	183	4295.09	0	31.20	33.16	1.96	1.06
**2.2**	30.9	0	72	2.17	63.12	183	4277.34	0	31.20	33.1	1.90	1.06
**2.3**	30.87	0	74	2.86	63	179	4248.86	0	31.20	33.1	1.90	1.06
**2.4**	31.31	0	60	2.78	50.09	98	4310.094	0	31.66	34.23	2.57	1.08
**2.5**	31.41	0	63	3.76	50.01	96	4273.99	0	31.66	33.93	2.27	1.07
**2.6**	31.07	0	68	5.61	50.05	96	4353.3	0	31.66	34.28	2.62	1.08
